# Amastin Knockdown in *Leishmania braziliensis* Affects Parasite-Macrophage Interaction and Results in Impaired Viability of Intracellular Amastigotes

**DOI:** 10.1371/journal.ppat.1005296

**Published:** 2015-12-07

**Authors:** Rita Marcia Cardoso de Paiva, Viviane Grazielle-Silva, Mariana Santos Cardoso, Brenda Naemi Nakagaki, Rondon Pessoa Mendonça-Neto, Adriana Monte Cassiano Canavaci, Normanda Souza Melo, Patrícia Massara Martinelli, Ana Paula Fernandes, Wanderson Duarte daRocha, Santuza M. R. Teixeira

**Affiliations:** 1 Departamento de Bioquímica e Imunologia, Universidade Federal de Minas Gerais, Belo Horizonte, Minas Gerais, Brazil; 2 Departamento de Parasitologia, Universidade Federal de Minas Gerais, Belo Horizonte, Minas Gerais, Brazil; 3 Faculdade de Farmácia, Universidade Federal de Minas Gerais, Belo Horizonte, Minas Gerais, Brazil; 4 Departamento de Bioquimica e Biologia Molecular, Universidade Federal do Paraná, Curitiba, Paraná, Brazil; 5 Departamento de Morfologia, Universidade Federal de Minas Gerais, Belo Horizonte, Minas Gerais, Brazil; Washington University School of Medicine, UNITED STATES

## Abstract

Leishmaniasis, a human parasitic disease with manifestations ranging from cutaneous ulcerations to fatal visceral infection, is caused by several *Leishmania* species. These protozoan parasites replicate as extracellular, flagellated promastigotes in the gut of a sandfly vector and as amastigotes inside the parasitophorous vacuole of vertebrate host macrophages. Amastins are surface glycoproteins encoded by large gene families present in the genomes of several trypanosomatids and highly expressed in the intracellular amastigote stages of *Trypanosoma cruzi* and *Leishmania* spp. Here, we showed that the genome of *L*. *braziliensis* contains 52 amastin genes belonging to all four previously described amastin subfamilies and that the expression of members of all subfamilies is upregulated in *L*. *braziliensis* amastigotes. Although primary sequence alignments showed no homology to any known protein sequence, homology searches based on secondary structure predictions indicate that amastins are related to claudins, a group of proteins that are components of eukaryotic tight junction complexes. By knocking-down the expression of δ-amastins in *L*. *braziliensis*, their essential role during infection became evident. δ-amastin knockdown parasites showed impaired growth after *in vitro* infection of mouse macrophages and completely failed to produce infection when inoculated in BALB/c mice, an attenuated phenotype that was reverted by the re-expression of an RNAi-resistant amastin gene. Further highlighting their essential role in host-parasite interactions, electron microscopy analyses of macrophages infected with amastin knockdown parasites showed significant alterations in the tight contact that is normally observed between the surface of wild type amastigotes and the membrane of the parasitophorous vacuole.

## Author Summary

Leishmaniasis is a parasitic disease caused by more than 20 species of the genus *Leishmania* that affects about 12 million people throughout the world and for which there is not an effective vaccine. Depending on the *Leishmania* species, clinical manifestation of the disease varies from self-resolving skin lesions to life-threatening visceralizing diseases. In addition to the toxicity of currently available drugs, their long treatment course, and limited efficacy, a major concern is the development of drug resistant parasite and more virulent variants. Together with the urgent need to develop new drugs that are more effective against this parasite as well as a vaccine to prevent new infections, it is also imperative to develop a better understanding of the factors that determine *Leishmania* virulence. Here, we describe the characterization of a gene family encoding surface proteins preferentially expressed in the mammalian stage of *Leishmania* that may be directly involved with the close interaction that is established between the intracellular parasite and host cell membranes. By inhibiting amastin gene expression in *L*. *braziliensis* in a mouse model of infection, we showed that these proteins are essential for intracellular parasite survival.

## Introduction

More than 20 species of the genus *Leishmania* cause leishmaniasis, a human illness with a large spectrum of clinical manifestations that range from self-resolving skin lesions to life-threatening visceral diseases. Endemic in eighty-eight countries from tropical and subtropical areas of the world, *Leishmania*sis has an estimated prevalence of 12 million cases with annual mortality rate of 60,000 people (www.who.int/topics/leishmaniasis/en/) for which there is no vaccine or adequate treatment. Thus, studies of various *Leishmania* species including the complete genome sequences of *Leishmania major*, *Leishmania infantum* and *Leishmania braziliensis* [1, 2] have been directed towards the identification of virulence factors used by the parasite to infect and survive within mammalian host cells as well as towards the development of new forms of treatment and disease prevention. Although comparative studies showed that *L*. *major*, *L*. *braziliensis* and *L*. *infantum* have very similar genomes regarding gene content and organization, the presence of specific sequences and pathways, such as retrotransposons and an active RNAi machinery found in *L*. *braziliensis* [3, 4], indicate a greater than expected diversity within this species. The differences regarding the presence of RNAi machinery also imply that different approaches must be used for functional genomic studies with these parasites.

During their life cycle, all *Leishmania* parasites alternate between the alimentary tract of a sandfly vector, where they grow as extracellular, flagellated promastigotes, before differentiating into infective non-dividing metacyclic forms and the phagolysosome of vertebrate host mononuclear phagocytes, where they multiply as amastigotes [5]. Therefore, the study of *Leishmania* proteins involved with the receptor-mediated phagocytosis and intracellular survival in the phagolysosome is critical for our understanding of leishmaniasis and the complex interaction between this parasite and its mammalian hosts.

One of the main characteristics of the genome of several members of the Trypanosomatid family is the presence of large numbers of repetitive sequences, especially multigene families encoding glycoproteins that are important components of the parasite surface directly involved in host-parasite interaction [6]. Among these multigene families are amastins, a group of surface glycoproteins containing 180–200 amino acids initially identified as a differentially expressed gene in *T*. *cruzi* amastigotes [7] as well as in *L*. *major* and *L*. *infantum* [8, 9]. More recently, genome data from various trypanosomatids showed that amastins are present in other *Leishmania* species as well as in *Crithidia* ssp. and *Leptomonas seymouri* [10–12], but no homologous sequences can be found outside the Trypanosomatid family. The predicted topologies of different amastin sequences in both *T*. *cruzi* and *Leishmania* spp. showed that all amastin proteins contain four hydrophobic transmembrane domains, interspersed with two serine and threonine rich extracellular domains and N- and C- terminal tails facing the cytosol. Comparative sequence analyses of different amastin genes have also indicated that, although the hydrophilic extracellular domain contains a conserved amastin signature (C-[IVLYF]-[TS]-[LF]-[WF]-G-X-[KRQ]-X-[DENT]-C), its sequences display significantly higher variability compared to hydrophobic domain sequences [9, 13]. Recent analyses of the evolution and diversification of amastin genes indicate that this family, which has undergone a major diversification after the genus *Leishmania* originated, can be grouped into four subfamilies, α, β, γ and δ-amastins, according to genomic position, structure and evolution [12]. Based on phylogenetic inferences and on the fact that the δ-amastin gene repertoire has been largely expanded in all *Leishmania* species, it has been suggested that the amastigote-specific function of amastins may possibly be limited to δ-amastins. It is noteworthy that δ-amastins are present in *T*. *cruzi* but absent in *T*. *brucei* and in other salivarian trypanosomes that do not have an intracellular stage, further suggesting that the expansion of δ-amastin genes in *T*. *cruzi* and *Leishmania* spp may be associated with the adaptation of amastigotes to the intracellular life stage [14].

With 45 copies identified in the genomes of *L*. *major* and *L*. *infantum* [12], amastins constitute the largest gene family in the *Leishmania* genus whose members show regulated expression during the life cycle of the parasite. *Leishmania* amastins are among the most immunogenic of all the surface antigens in mice [15] and elicit a strong immune response in humans, particularly associated with visceral leishmaniasis [16]. With only 12 copies of δ-amastins, *T*. *cruzi* has a more limited amastin gene repertoire [7, 17]. Besides δ-amastins, *T*. *cruzi* has two copies of β-amastins, which, surprisingly, have been found to be upregulated in the insect, epimastigote forms of the parasite [18].

Although it has been more than 20 years since their discovery, the function of amastins remains unknown. The fact that they are encoded by a family with a large number of copies in the genomes of different trypanosomatids that have an intracellular stage and are located on the surface of the parasites led us to infer that these proteins may interact with molecules from the host cell or act as membrane transporters. Since gene knockout is not an option, due to the large number of amastin genes, RNA interference (RNAi) knockdown constitutes the best strategy to address gene function in diploid pathogenic organisms that have no defined sexual cycle. First described in 1998, RNAi has quickly proven to be an immensely useful tool for studying gene function in *T*. *brucei* [19]. However genome data has shown that the components of the RNAi machinery are absent in *T*. *cruzi* [20], *Leishmania major*, *L*. *donovani* [21, 22] as well as in other protozoan parasites such as *Plasmodium falciparum* [23]. Unexpectedly, in 2010 Lye and colleagues demonstrated that *L*. *braziliensis* and other species of the sub-genus *Viannia* have an active RNAi machinery [4]. Although these authors have clearly demonstrated that RNAi activity in *L*. *braziliensis* results in down-regulation of the expression of a reporter gene such as the green fluorescent protein (GFP) gene as well as endogenous genes, such as the paraflagellar rod protein, no other study aimed at investigating gene function in *L*. *braziliensis* using RNAi has been described.

Here we present data on the sequence and structural organization of amastin genes in *L*. *braziliensis* and show that α, β, γ and δ-amastins are upregulated in *L*. *braziliensis* amastigotes and are localized to the parasite surface. We also show that RNAi knockdown of δ-amastin expression in *L*. *braziliensis* results in decreased survival and proliferation of intracellular parasites after either *in vitro* infection of mouse macrophages or *in vivo* infection of mice. These results, together with observations from ultrastructural analysis, which show significant alterations in the membrane contact between host macrophage and intracellular amastigotes, indicate that *Leishmania* amastins are virulence factors essential for parasite replication within the mammalian host cell.

## Results

### Genomic organization and structural analysis of amastin genes in *L*. *braziliensis*

**Fig 1:**
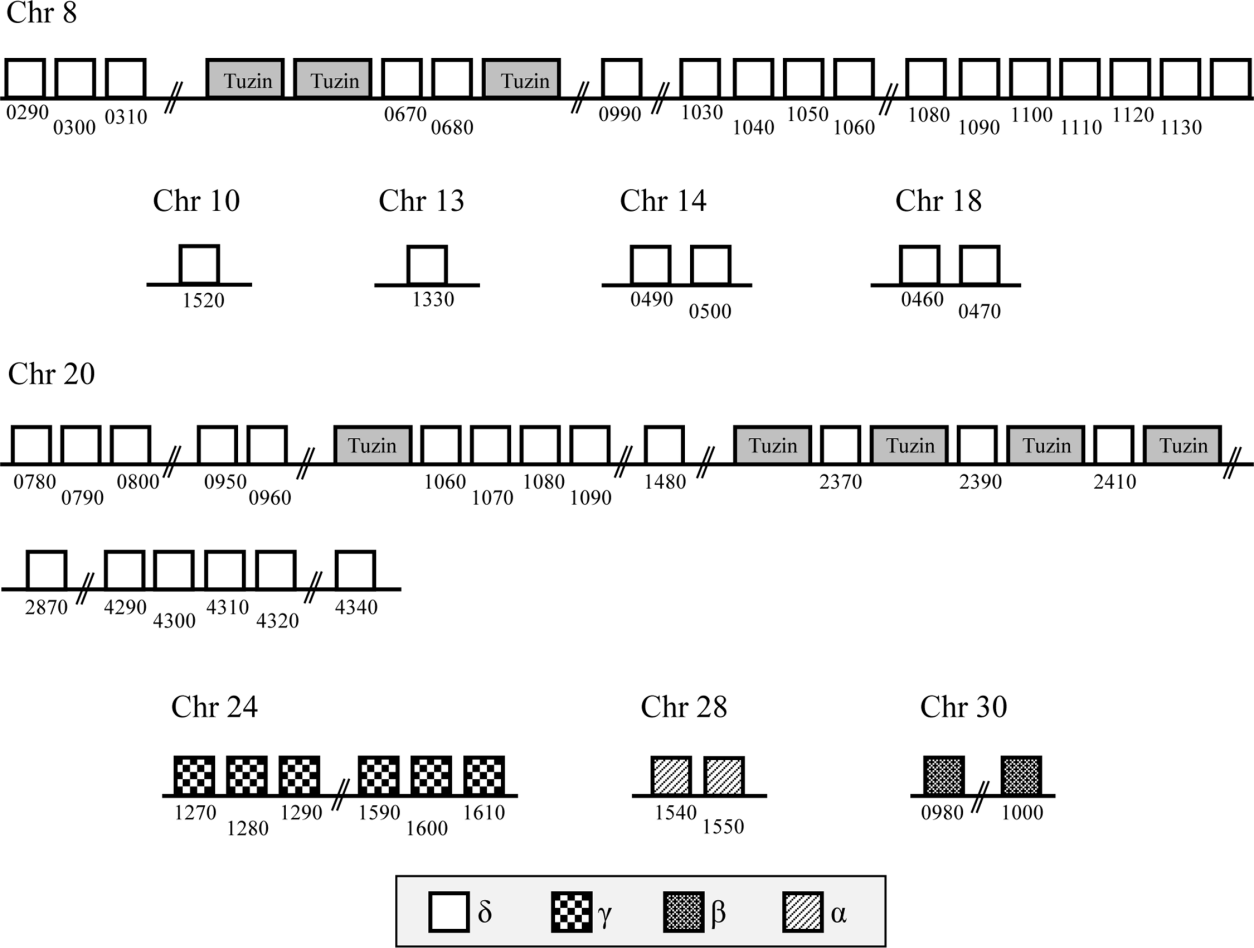
Genomic organization of the amastin genes in *L*. *braziliensis*. The schematic representation of 52 amastin gene copies located on nine different chromosomes was based on the complete genome sequence of *L*. *braziliensis* MHOM/BR/75/M2904 obtained from the Tritryp database (www.tritrypdb.org). The numbers below the boxes correspond to the given names of each amastin *L*.*braziliensis* gene homolog in GeneDB. Boxes with the same background correspond to amastin gene copies belonging to the same subfamily. Eight tuzin genes, shown as gray boxes, are associated only with δ-amastins.

**Fig 2:**
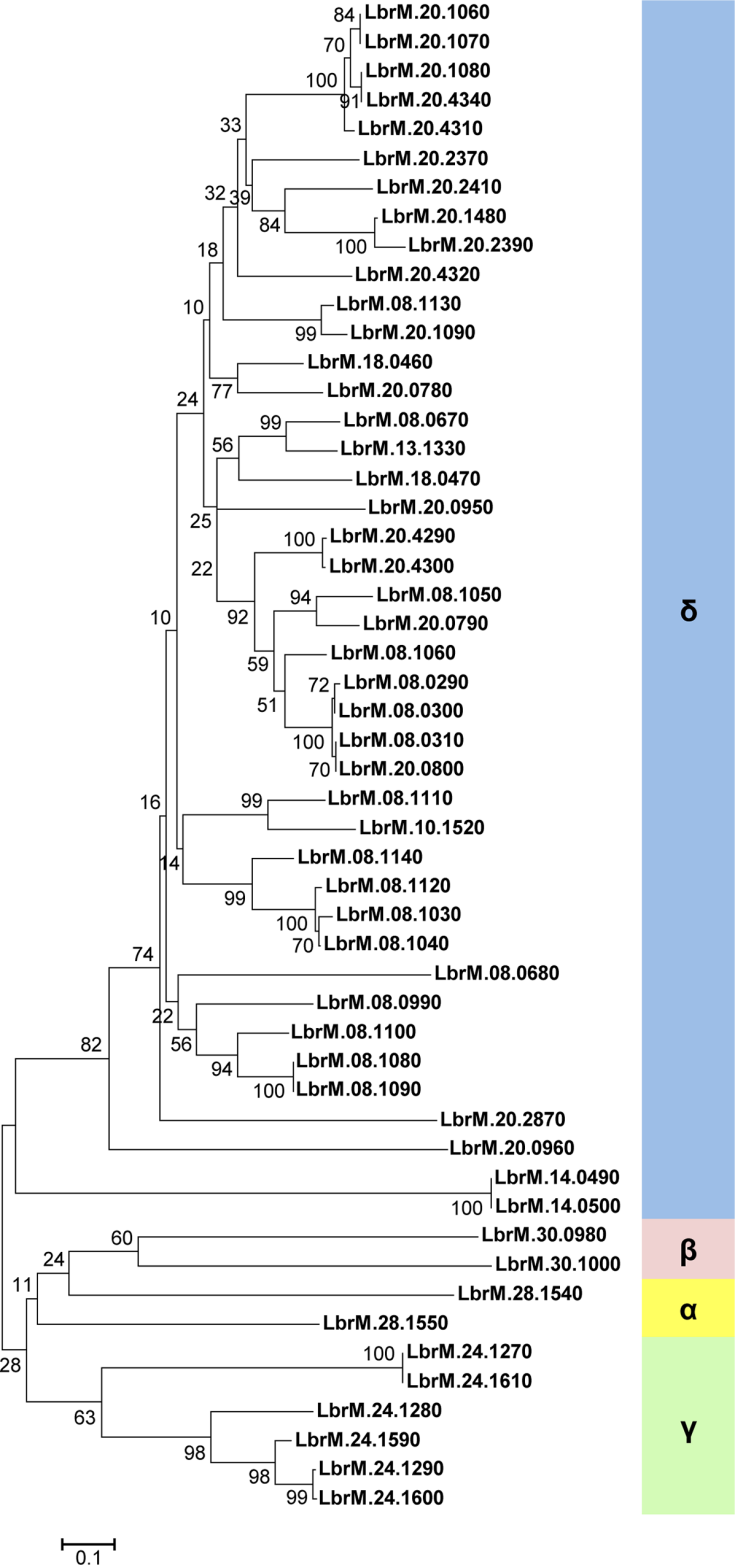
Phylogenetic tree of amino acid sequences of 52 amastins from *L*. *braziliensis*. The sequences were aligned using the MUSCLE algorithm and a neighbor joining tree was generated using the MEGA6 software. Branch lengths are drawn proportion to evolutionary change with bootstrap values shown on each node. Classification into four amastin sub-families shown on the right was based on Jackson (2010).

**Fig 3:**
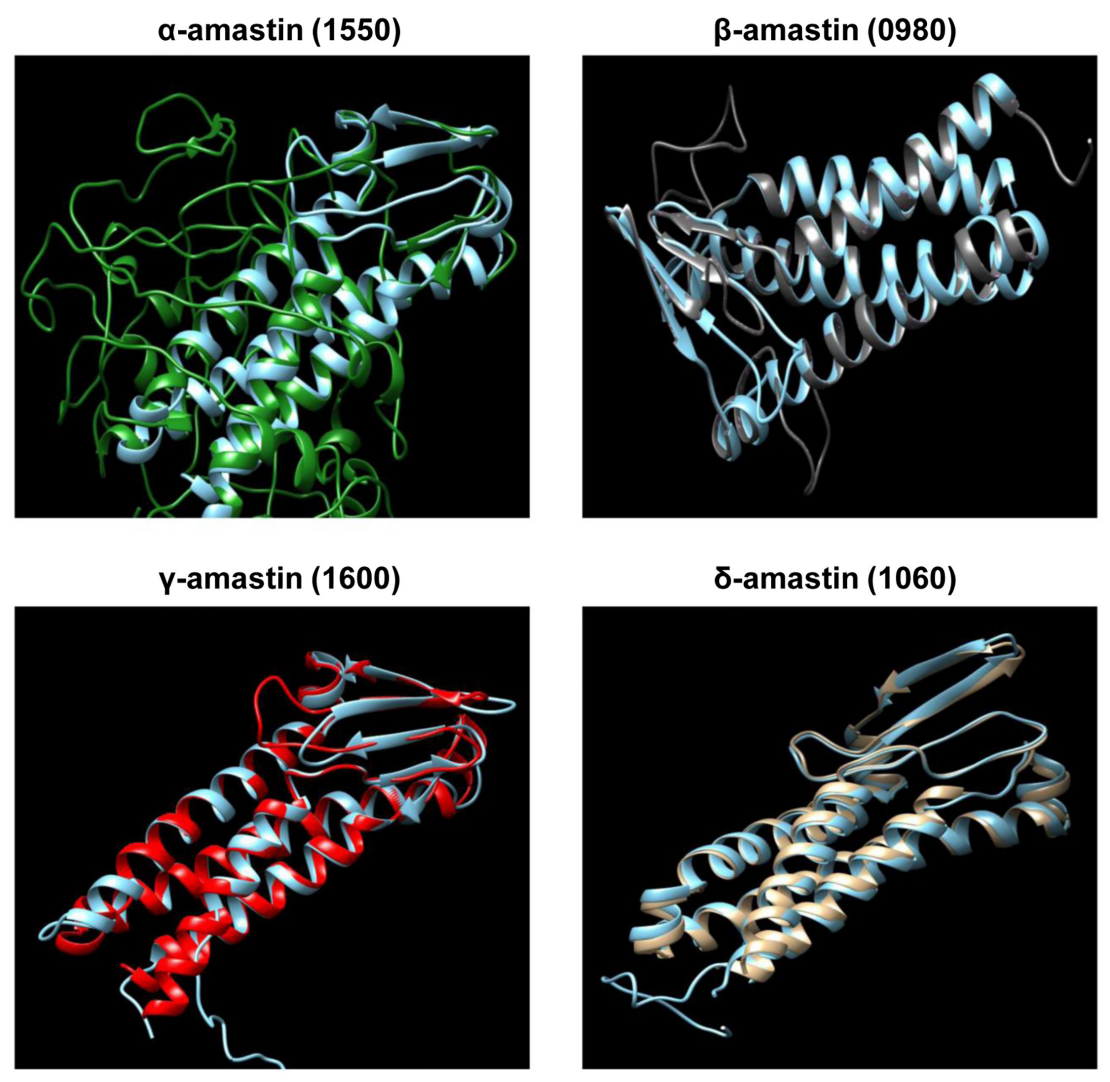
Homology-based 3D modeling of α, β, γ and δ amastins. Structural predictions were done using PHYRE web server and the predicted structures of α, β, γ and δ amastins, were imaged using the UCFS Chimera program. α-amastin is shown in green, β-amastin is shown in gray, γ-amastin is shown in red, δ-amastin is shown in yellow and the superimposed mouse claudin 15 model is shown in blue.

To identify all protein coding genes present in *L*. *braziliensis* with sequence similarity to amastins, we screened the *L*. *braziliensis* genome database (www.tritrypdb.org) for sequences homologous to *T*. *cruzi* and *L*. *infantum* amastins. A total of 52 genes, located on nine different chromosomes and belonging to all four previously described amastin sub-families, namely α, β, γ and δ-amastins, were identified (Fig 1). Similar to the amastin gene family organization found in the genomes of *T*. *cruzi* and *L*. *infantum* [7, 9], only δ-amastins were found associated with tuzin gene orthologs. A phylogenetic tree based on amino acid sequences of all 52 *L*. *braziliensis* amastin sequences shows three major clusters with α and β-amastins clustered together and γ and δ-amastins showing highly divergent sequences (Fig 2). Fig 2 also shows that, similar to other *Leishmania* species, such as *L*. *infantum*, *L*. *major* and *L*. *amazonensis*, *L*. *braziliensis* has a diverse amastin gene repertoire and that its expansion derives from the expansion of the δ-amastin subfamily. Multiple sequence alignments of amastin-related sequences from *L*. *braziliensis*, *L*. *infantum* and *T*. *cruzi* demonstrated significant conservation both in sequence and in the predicted structures amongst the different family members. The four predicted transmembrane helices are highly conserved and show high sequence similarity amongst the amastin family members (S1 Fig). Similar to *T*. *cruzi* δ-amastins [13], the two predicted extracellular, hydrophylic domains, have increased sequence variability compared to the transmembrane domains. Also similar to *T*. *cruzi* and *Leishamania* spp amastins, a consensus sequence, previously identified in *L*. *infantum* amastins as the amastin signature [9], is also found in all *L*. *braziliensis* sequences (S1 Fig). Since primary sequence alignments showed no homology to any known protein sequence, we used the protein fold recognition server Phyre (Protein Homology Recognition Engine) [24] to generate a predicted structural model for amastins. Homology modeling indicated that α, β, γ and δ-amastins are related to a group of proteins that are components of tight junction complexes named claudins. Claudins constitute a protein family of 27 members in mammals, with a molecular mass ranging from 20 to 27 kDa [25]. Fig 3 shows that α, β, γ and δ-amastins have significant structural homology with the predicted 3D structures of claudin 15. Similar to amastins, claudins bear four transmembrane domains, a short intracellular N-terminal sequence, a large first extracellular loop (~50 residues), a shorter second extracellular loop (16–33 residues) and a cytoplasmic domain of variable length [26]. Members of the claudin protein family also display conserved residues in the first extracellular domain (W-LW-C-C) [27] that partially matches the amastin signature (F/W-LW-C-C) described by Rochette *et al*. (2005) [9].

**Fig 4:**
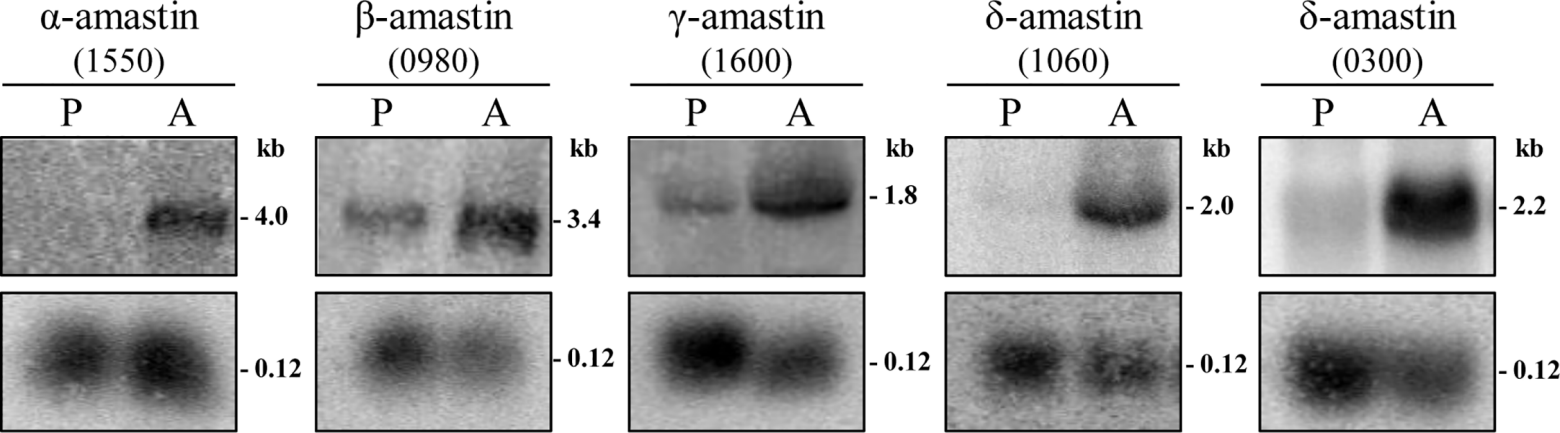
Differential expression of amastin mRNA during the *L*. *braziliensis* life cycle. Total RNA (10 μg/lane), extrated from promatigote (P) and axenic amastigote (A) forms were separated by electrophoresis, transfered to nylon membranes and probed with the ^32^P-labelled sequences corresponding to an α-amastin (LbM.28.1550), β-amastin (LbM.30.0980), γ-amastin (LbrM.24.1600) and two δ-amastins (LbM.08.0300 and LbrM.20.1060). Bottom panels show hybridization of the same membranes with a fragment of the 5S rRNA.

Although amastin genes have been initially described as amastigote specific genes, we have recently demonstrated that, in contrast to δ-amastins transcripts, transcript levels of *T*. *cruzi* β-amastins are upregulated in epimastigotes, the stage found in the triatomine vector [18]. Here we determined steady state levels of amastin transcripts belonging to the four sub-families identified in the *L*. *braziliensis* genome using total RNA extracted from promastigotes and axenically growing amastigotes. Northern blots probed with radiolabelled fragments containing sequences corresponding to one member of α, β and γ-amastins (LbrM.28.1550, LbrM.30.0980 and LbrM.24.1600, respectively) and two members of δ-amastins, (LbrM.20.1060, LbrM.08.0300), showed that transcript levels of amastins belonging to all four subfamilies are upregulated in the amastigote stage compared to the promastigote stage (Fig 4). RNA quantification using rRNA as a loading control showed the level δ-amastin transcripts was increased by 19-fold in amastigotes compared to promastigotes, whereas α, β and γ-amastin transcripts were increased only 3, 2 and 7-fold, respectively, when comparing amastigotes to promastigotes.

### *L*. *braziliensis* amastin proteins are localized at the parasite surface

**Fig 5:**
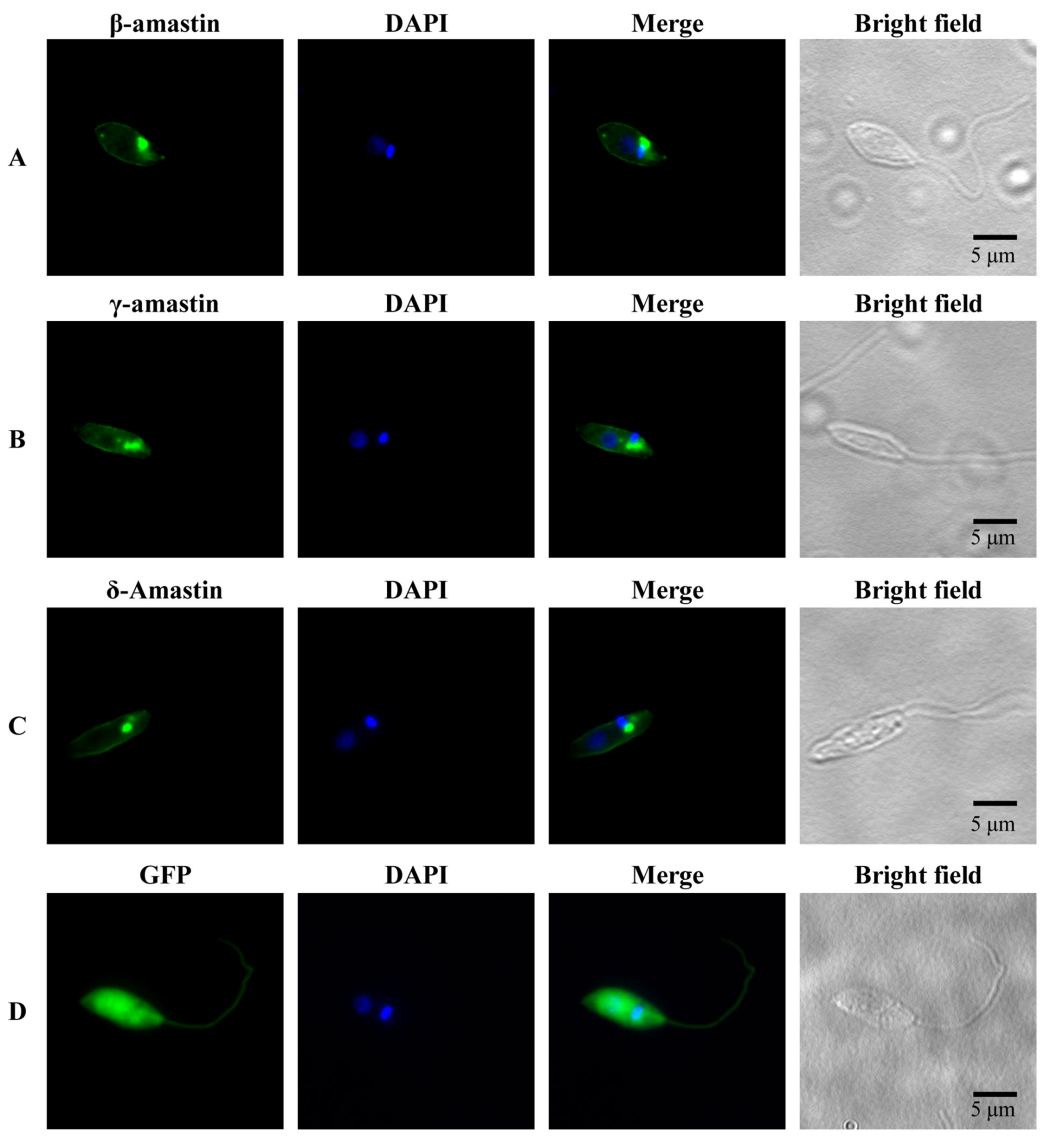
Subcellular localization of distinct amastins in fusion with GFP. Promastigotes were transiently transfected with the plasmids pSP-Ama0980-GFP (A), pSP-Ama1600-GFP (B), pSP-Ama1060-GFP (C) and pSP-nGFP (D) as a control plasmid. Transfected parasites were fixed with 2% paraformaldehyde, stained with DAPI and visualized under a fluorescence microscope. Nuclear and kinetoplast DNA are shown in blue.

As shown by Rochette *et al*. [9] transfection of *L*. *infantum*, *L*. *major* and *L*. *infantum* with vectors containing amastin sequences fused to green fluorescent proteins results in parasites with fluorescent signals in their plasma membranes. Similarly, *T*. *cruzi* epimastigotes and amastigotes transfected with GFP-fusion constructs of β and δ-amastins [18], as well as immune-electron micrographs of amastigotes using an anti- δ-amastin peptide antibody [7] showed a surface localization of the protein. As indicated before, hydrophobicity profiling predicted four transmembrane helices for all *L*. *braziliensis* amastin homologs tested (S1 Fig), thus suggesting that similar to *T*. *cruzi* amastins and other *Leishmania* amastin homologs, *L*. *braziliensis* amastins have a surface localization in the parasite. To verify this, we prepared GFP fusion constructs of three distinct amastin sequences in the expression vector pSPGFP [28] and transfected *L*. *braziliensis* promastigotes. As shown in Fig 5, expression of GFP fusion proteins containing sequences of β, δ and γ-amastins resulted in parasites showing fluorescence signals in the plasma membrane but excluded from the flagellum. For reasons that are unknown, several attempts to obtain the expression of α-amastin fused to GFP have failed.

### Generation of *L*. *braziliensis* cell lines with amastin expression knocked-down by RNAi

**Fig 6:**
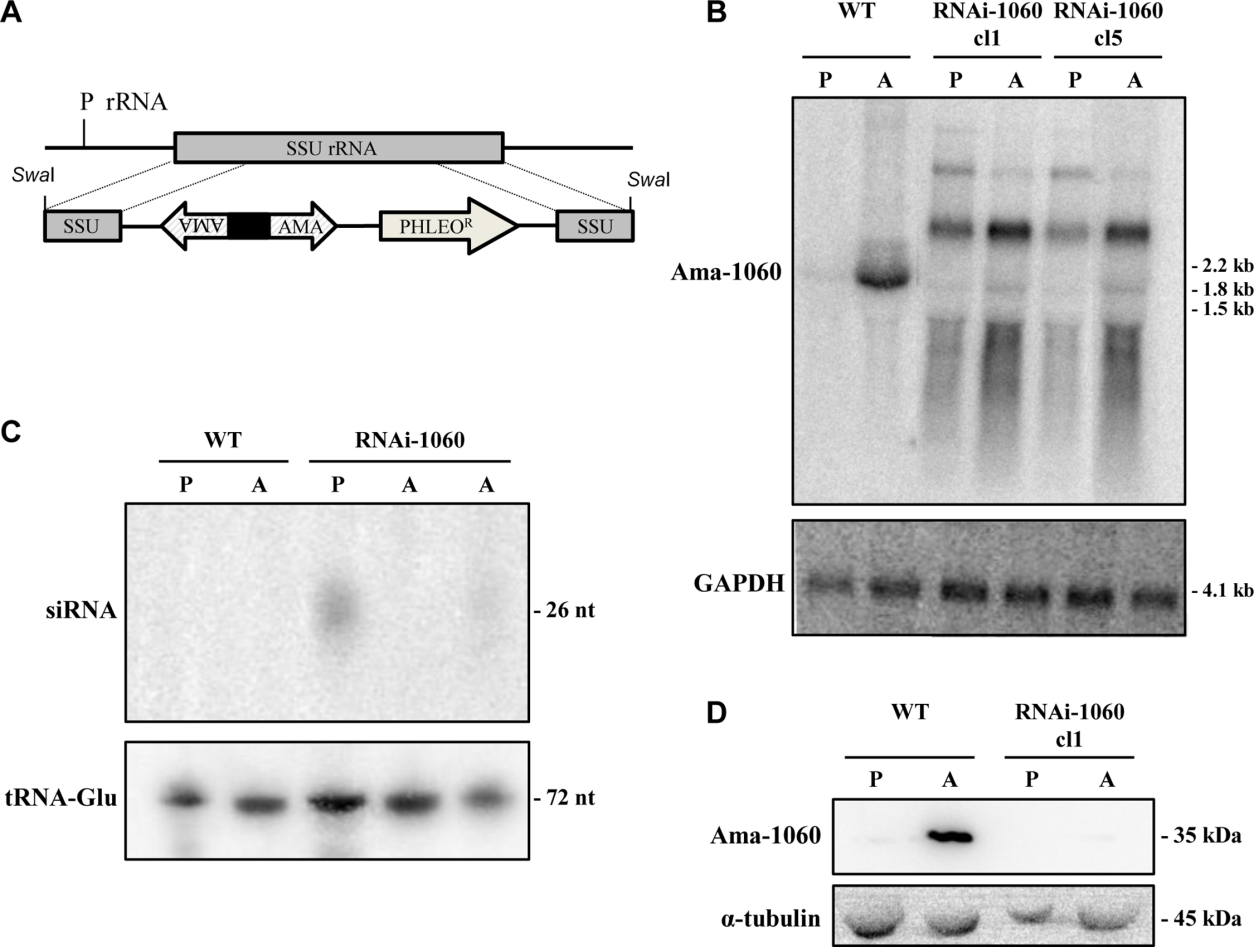
RNAi knockdown of amastin genes in *Leishmania braziliensis*. (A) The plR1-Phleo plasmid containing two opposite amastin fragments with a stem-loop stuffer fragment (black box), Phleomycin resistance (gene PHLEO) and the rRNA promoter (P rRNA) is shown integrated into the SSU rRNA locus of *Leishmania* (gray box). (B) Northern blot analyses of RNA isolated from (P) promastigote and (A) axenic amastigotes from wild type *L*. *braziliensis* (WT) and two cloned cell lines of *L*. *braziliensis* transfected with a construct that generates δ-amastin dsRNA named RNAi-1060-cl1 and cl5. The blots were probed with a ^32^P-labelled DNA fragment corresponding to the LbrM.08.1060 amastin gene. (C) Low-molecular-weight RNAs isolated from promastigotes and amastigotes from WT *L*. *braziliensis* as well as from promastigotes from RNAi-1060 cl1 and amastigotes from the two cloned cell line transfected with amastin dsRNA constructs (RNAi-1060 cl1 and cl5) were fractionated on a 15% polyacrylamide gel and probed with a mixture of ^32^P-labelled oligonucleotide probes corresponding to the full length LbrM.08.1060 amastin gene. siRNA indicates the position of small interfering RNA bands that hybridized with δ-amastin oligonucleotide probes, which co-migrate with a 26 nt DNA molecular weight marker. Hybridization of the same blot with a probe corresponding to the *L*. *braziliensis* Glu-tRNA is also shown as a loading control. (D) Total protein extracts from the cloned cell RNAi-1060 cl1 was analyzed by western blot using an antibody generated against the recombinant Ama1060. The same blot was incubated with anti-α-tubulin as a loading control.

The genome sequence of *L*. *braziliensis* predicts the existence of an active RNAi pathway [2] and this prediction has been experimentally validated [4]. To further investigate the role of amastin genes, we knocked-down their expression in *L*. *braziliens*is by transfecting promastigotes with the pIR1PHLEO vector containing sense and anti-sense coding sequences of the δ-amastin gene LbrM.20.1060 (Fig 6A). To confirm that amastin gene expression has been knocked down, we isolated RNA from two cloned transfected cell lines and probed northern blot with labelled LbrM.20.1060 DNA fragment. As shown in Fig 6B, amastin transcripts were degraded in amastigotes derived from the two cloned cell lines that have been transfected with amastin dsRNA constructs, named RNAi1060-cl1 and RNAi1060-cl5. The same northern blot showed a 19-fold increase in amastin mRNA levels in WT, untransfected amastigotes compared to promastigotes, as previously described. Northern blot analysis also showed that mRNA degradation occurs specifically with the amastin mRNA since high levels of full length GAPDH mRNAs were detected in all cell lines. High molecular weight bands observed in the RNA samples purified from RNAi knockdown mutants may correspond to intact or partially fragmented double stranded, stem-loop RNA, as previously detected by Lye *et al*. (2010) in *L*. *braziliensis* expressing stem-loop dsRNA with sequences from the GFP gene or from genes encoding enzymes for lipophosphoglycan synthesis [4]. To verify that the mRNA degradation is due to the presence of siRNAs in the transfected parasites, we purified small-molecular-weight RNAs from WT and from two Ama1060-RNAi clones, fractionated them on a 15% polyacrylamide gel and probed with labelled 60 oligonucleotides containing sequences spanning almost the entire coding region of amastin gene LbrM.20.1060. Fig 6C shows that siRNAs with about 26 nt are detected in the RNA population of promastigotes derived from one of the transfected *L*. *braziliensis* cell lines, whereas only weak signals were detected in amastigotes derived from the two transfected, Ama1060-RNAi clones. To confirm that the RNAi machinery has efficiently knocked down amastin protein expression in *L*. *braziliensis*, western blots with total protein extracts were incubated with antibodies made against the recombinant LbrM.20.1060 protein. Fig 6D shows that whereas in WT parasites the expression of amastin was found exclusively in amastigotes, protein levels of amastins in the transfected cell lines are below the detection limit of the western blot. Knockdown of δ-amastin expression did not affect growth of promastigote mutants or differentiation into metacyclic forms in stationary phase promastigote cultures.

To determine whether siRNA containing sequences derived from one amastin gene (LbrM.20.1060) affected the expression of additional members of the amastin gene familily, we performed a genome wide sequencing analysis of polyA+ RNA purified from amastigotes from WT *L*. *braziliensis* and from the one RNAi amastin knockdown cell line. As shown in S2 Fig and S1 Table, expression of dsRNAs derived from one specific δ-amastin gene (LbrM.20.1060) affected transcript levels of most members (60%) of the δ-amastin subfamily but did not affect transcript levels of α, β and γ-amastins. In addition, RNAi knockdown of δ-amastin does not affect the expression of other *L*. *braziliensis* genes, such as *gapdh*.

### δ-amastin knockdown results in decreased numbers of intracellular amastigote in *in vitro* macrophage infection experiments and no detectable parasites after *in vivo* infection

**Fig 7:**
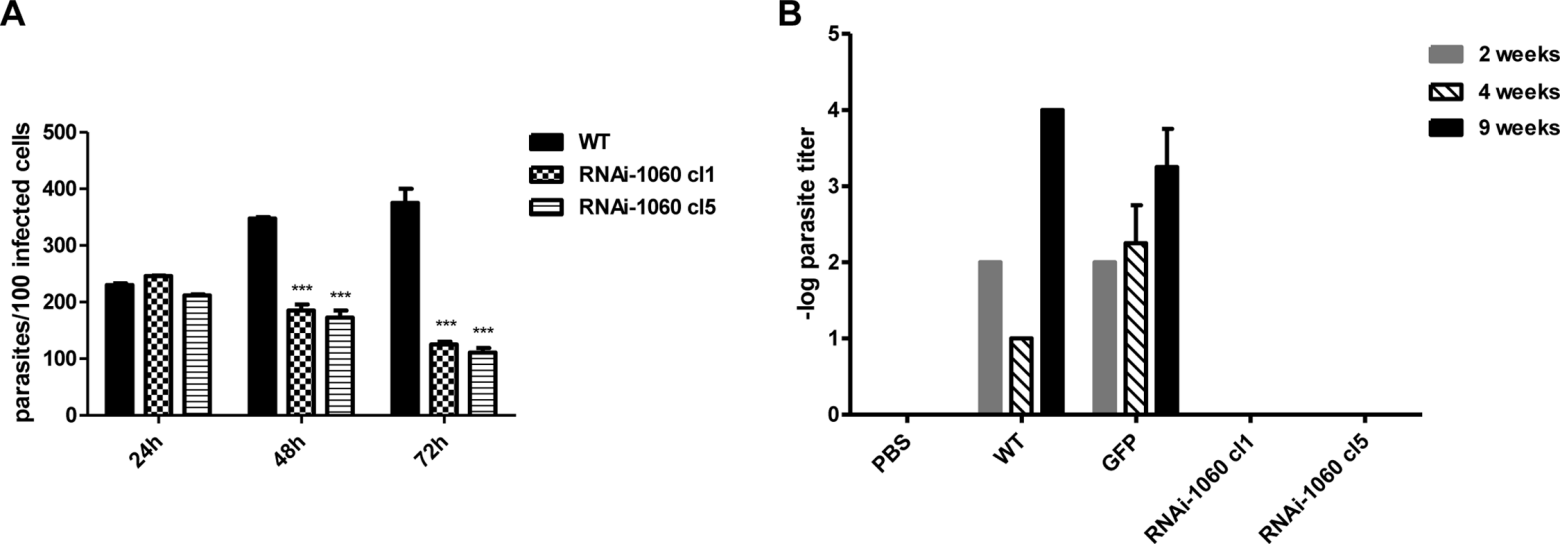
Infection of mouse macrophages and BALB/c mice footpads with WT *L*. *braziliensis* and RNAi-1060 cell lines. (A) Intraperitoneal macrophages from BALB/c mice were incubated for 24 hours at 34°C with stationary phase promastigotes from WT *L*. *braziliensis* cultures and the two cloned cell lines RNAi-Ama1060 cl1 and cl5 at a ratio of 10: 1 parasites/cell. After washing non-internalized promastigotes macrophages were incubated for 24, 48 or 72 hours before the cells were stained with DAPI and the numbers of intracellular amastigotes, visualized by fluorescence microscopy, were determined. (B) BALB/c mice were infected in the footpads with 10^7^ stationary phase promastigotes from WT *L*. *braziliensis*, *L*. *braziliensis* transfected with the pIR1PHLEO vector containing GFP, and two cloned cell lines expressing δ-amastin siRNA, RNAi-1060cl1 and cl5. Two, four and nine weeks after infection, parasitism was evaluated by the limiting dilution method.

To verify whether RNAi-mediated depletion of δ-amastin mRNA affected parasite infection capacity, intraperitoneal macrophages were isolated from BALB/c mice and were incubated for 24 hours with either wild type (WT) *L*. *braziliensis* stationary phase promastigotes or promastigotes derived from two parasite cell lines with reduced expression of δ-amastins. After washing non-internalized parasites, the numbers of amastigotes per 100 cells were assessed 24, 48 and 72 hours post-infection. As shown in Fig 7A, no significant differences in the numbers of intracellular amastigotes were observed 24 hours after the infection, indicating that the initial steps of the *in vitro* infection were not affected by knocking down δ-amastin expression. However, compared to WT parasites, the number of intracellular amastigotes showed an average 3 fold reduction in cells infected with the two cloned cell lines expressing amastin dsRNA at 48 and 72 hours post-infection, suggesting that δ-amastin expression is required for parasite intracellular multiplication.

Next, we assessed the effect of knocking down δ-amastins in an *in vivo* model of infection by inoculating the footpad of BALB/c mice with equal numbers of stationary-phase promastigotes of (1) WT *L*. *braziliensis*, (2) a *L*. *braziliensis* cell line that has been transfected with the pIR1PHLEO vector containing the GFP gene or (3) the two cloned cell lines expressing δ-amastin dsRNA. By monitoring the infection through determination of parasite numbers in the infected animals, we verified that the expression of δ-amastins is essential for parasite survival in BALB/c mice. The results of three independent experiments clearly showed that whereas similar numbers of amastigotes were found in the tissues of mice infected with WT and with transfected, GFP-expressing *L*. *braziliensis*, no parasites could be recovered from tissues of mice inoculated with the two cloned, amastin knockdown cell lines two weeks after inoculation. Similar results were observed when parasite numbers were determined four and nine weeks post infection (Fig 7B).

**Fig 8:**
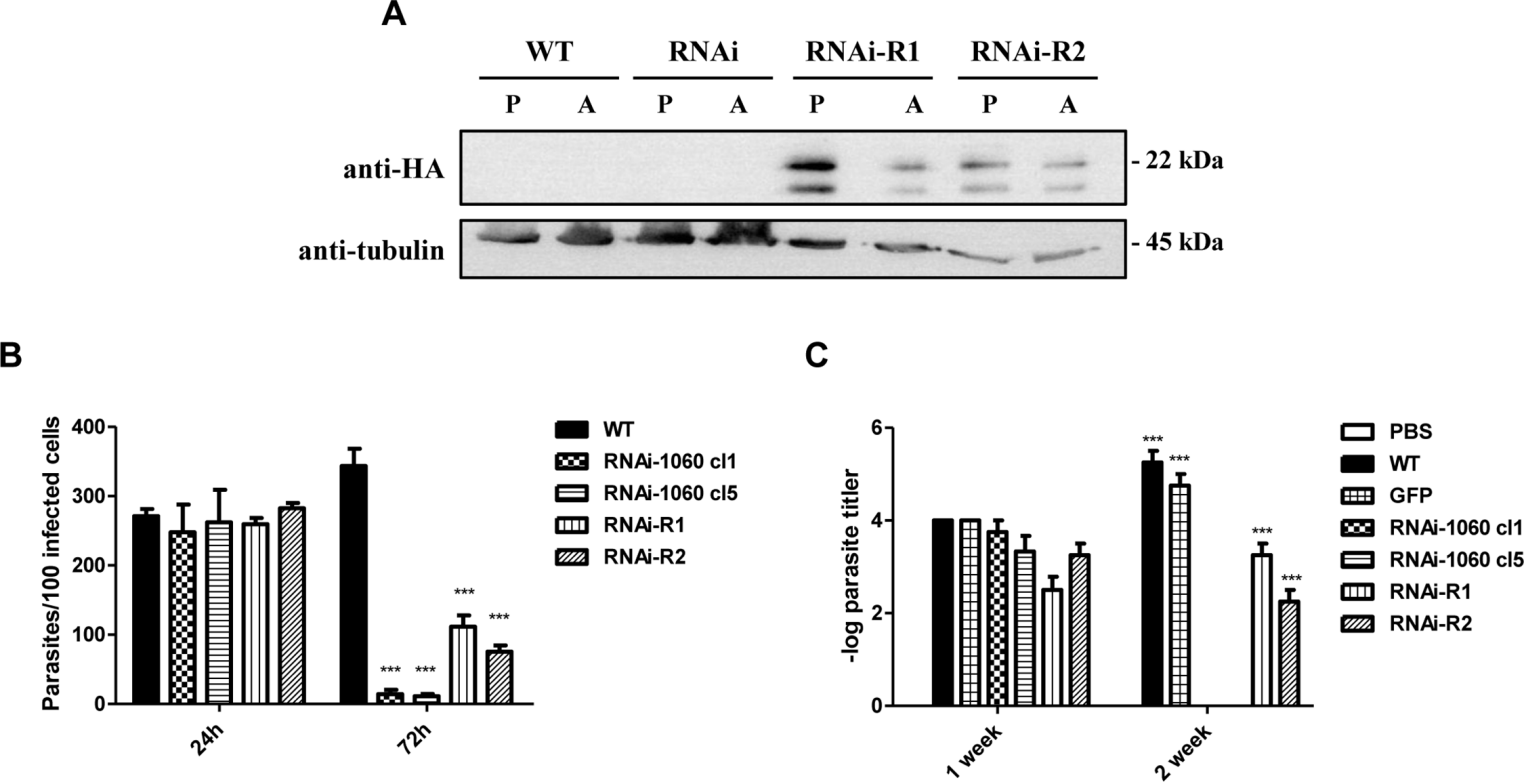
Re-expression of amastin sequences in RNAi knockdown parasites rescue infection capacity of *L*. *braziliensis*. (A) Total protein extracts from promastigotes (P) and amastigotes (A) of WT *L*. *braziliensis*, the cloned cell RNAi-1060 cl1 and two cloned cell lines derived from the RNAi-1060 parasites the were transfected with an RNAi-resistant amastin gene (RNAi-1060-R2 and RNAi-1060-R4) were analysed by western blot using an anti-HA antibody. Bands corresponding to the Ama1060 synthetic gene containing the HA epitope are shown only in the re-expressor cell lines. The same blot was incubated with anti-tubulin antibody as a loading control. (B) Stationary phase promastigotes from WT *L*. *braziliensis*, two cloned cell lines expressing amastin dsRNA, RNAi-1060 cl1 and cl5 and two cloned cell lines that express a RNAi resistant amastin sequence were used to infect BALB/c mice peritoneal macrophages at a ratio of 10:1 (parasites/cell) and the numbers of intracellular amastigotes determined 24 and 72 hours post-infection. (C) Stationary phase promastigotes (10^7^ parasites) from WT *L*. *braziliensis*, *L*. *braziliensis* transfected with the pIR1PHLEO vector containing GFP, the two cloned cell lines expressing amastin dsRNA, RNAi-1060 cl1 and cl5 and two cloned cell lines that express a RNAi resistant amastin sequence were used to infected BALB/c mice footpads. One or two weeks post-infection, parasitism was evaluated by the limiting dilution of parasites recovered from the mice footpads.

To confirm that such strong, attenuated phenotype was not a consequence of altered expression of genomic sequences other than δ-amastins, we set out to rescue this phenotype by expressing an RNAi-resistant amastin construct in one of the amastin knock down cell lines. A plasmid construct carrying a synthetic δ-amastin sequence containing third base modifications in such a way that the mRNA sequence is divergent from the wild type LbrM.20.1060 gene but the amino acid sequence remains exactly the same (S3 Fig) was transfected into the amastin cell line RNAi1060-cl5. In addition, to verify that this synthetic gene was being expressed in the transfected cell lines that also express amastin dsRNA, an HA epitope tag was added in the region encoding the second hydrophilic, extracellular domain of the protein. As shown in Fig 8A, transfection of the RNAi1060-cl5 cell line with a plasmid containing the RNAi-resistant δ-amastin sequence resulted in parasite cell lines (named RNAi1060-re-expressors, RNAi1060-R1 and RNAi1060-R2) expressing tagged amastin proteins that are recognized by anti-HA antibodies in both promastigotes and amastigotes. Fig 8B showed that these two cloned cell lines that are re-expressing δ-amastin LbrM.20.1060 were able to survive in mouse macrophages almost as well as WT *L*. *braziliensis*. As described before, no differences in the number of intracellular amastigotes were detected in macrophages infected with WT and the two RNAi clones, RNAi1060-cl1, RNAi1060-cl5 24 hours after infection. Twenty-four hours post-infection, the two re-expressor clones, RNAi1060-R1 and RNAi1060-R2, also showed similar numbers of intracellular amastigotes compared to WT parasites. However, when intracellular amastigote numbers were determined 72 hours post-infection, a clear difference was observed between both re-expressor clones and the two RNAi clones: whereas almost no amastigotes were found in macrophages infected with RNAi1060-cl1 and RNAi1060-cl5, the number of amastigotes in cells infected with the two re-expressor clones, RNAi1060-R1 and RNAi1060-R2, reached 35% and 25%, respectively, of the numbers found in cells infected with WT parasites. Reversion of this attenuated phenotype is even more clearly observed during *in vivo* infection: whereas no parasites could be rescued from infected animals two weeks after inoculating the RNAi knockdown cell lines, infection with the two re-expressor clones resulted in parasite numbers corresponding to 61 e 42% of the numbers found in mice that were infected with WT *L*. *braziliensis* (Fig 8C). To verify that the cells expressing the RNAi-resistant transgene continue to express δ-amastin dsRNA, we PCR amplified DNA purified from WT and the two RNAi clones, RNAi1060-cl1, RNAi1060-cl5, as well as from two cloned cell lines derived from the RNAi1060-cl5 that were transfected with the RNAi-resistant construct. PCR amplifications using a forward primer annealing in the PHLEO resistance marker present in the pIR1PHLEO-Ama1060 plasmid and a reverse primer annealing in the SSU ribosomal locus of the *Leishmania* genome showed that both re-expressor clones retained the pIR1PHLEO-Ama1060 plasmid construct, indicating that the these cells continue to express the amastin dsRNA (S4 Fig). These results showed that it is possible to re-express δ-amastins in a parasite that had this gene knocked down by RNAi and, by doing so, the parasite regains the ability to infect and multiply in BALB/c mice. This observation adds support for a role of these surface proteins as a *L*. *braziliensis* virulence factor.

### δ-amastin knockdown interferes with amastigote interaction with the parasitophorous vacuole membrane of infected macrophage

**Fig 9:**
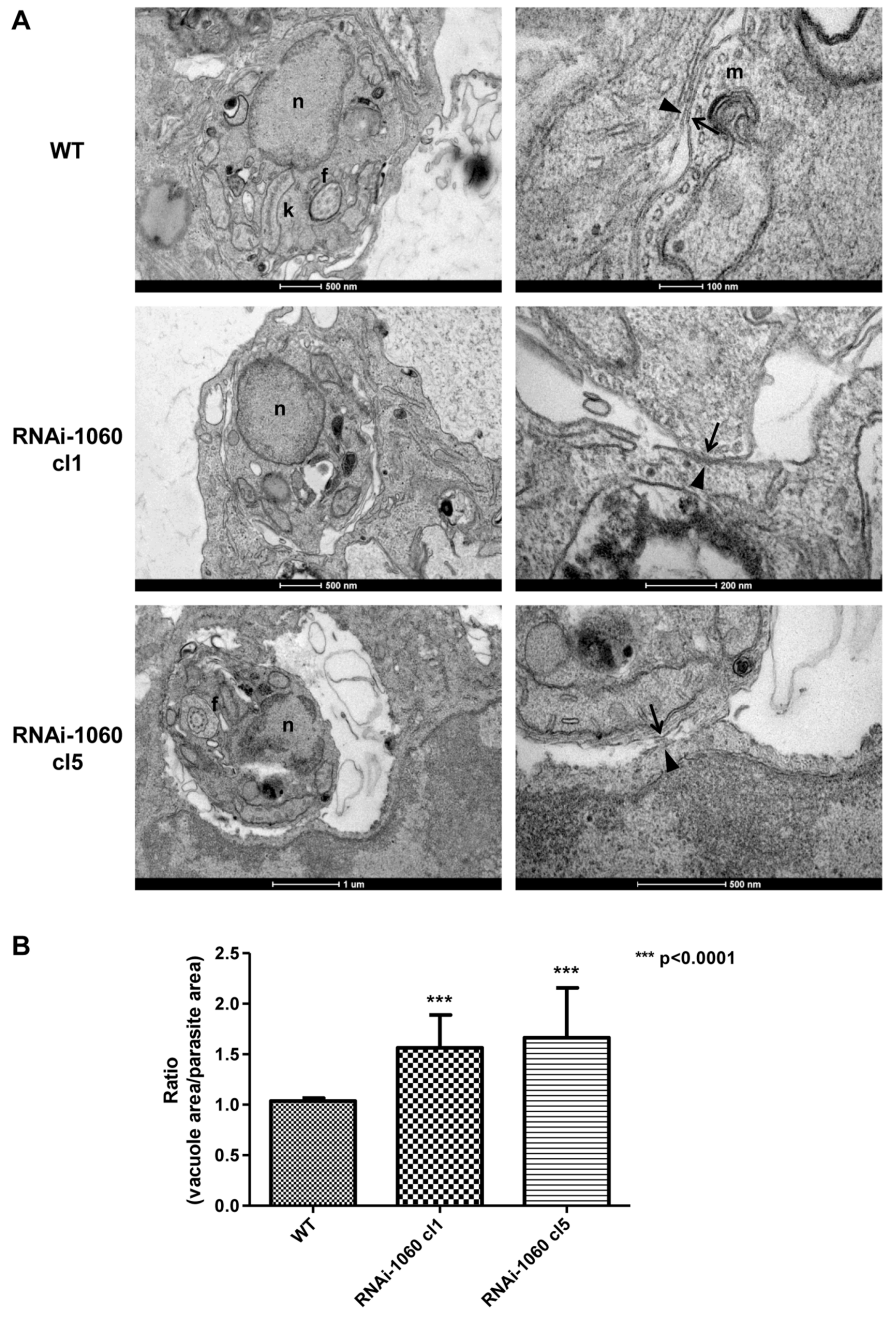
Amastin knockdown affects amastigote interactions with macrophage membranes. (A) Transmission electron microscopy of macrophages infected with WT *L*. *braziliensis* (WT) and two cloned cell lines expressing amastin siRNA, RNAi-1060 cl1 and RNAi-1060 cl5. Left side panels show amastigotes surrounded by the macrophage parasitophorous vacuole (PV) membrane. In contrast to WT parasites, that are in close contact with the PV membrane, larger distances between the membranes of intracellular amastigotes and the PV membranes are observed in macrophages infected with both clones of RNAi knockdown mutants. Right side panels show magnified images of the points of contact between parasite (arrows) and PV (arrowheads) membranes with regions where disrupted membrane structures are observed in macrophages infected with the RNAi knockdown mutants. Parasite nucleus (n), kinetoplast (k), subpellicular microtubule (m) and flagellum (f) are indicated by lower case letters. (B) Ploted ratios between the area of macrophage PV and the area of the amastigote inside each vacuole. Error bars represented the SD values obtained from measurements of the areas of twenty vacuoles and their respective parasites in each experimental group.

Macrophages infected with WT *L*. *braziliensis* as well as with δ-amastin knockdown parasite cell lines were further examined using transmission electron microscopy (TEM). As shown by Zauli *et al*. [29], TEM of macrophages infected with *L*. *braziliensis* allowed the identification of amastigotes exhibiting their characteristic subpellicular microtubules, kDNA structure and a short flagellum inside tight parasitophorous vacuoles (PV) (Fig 9A). Our TEM analyses also showed the existence of a tight interaction between the amastigote membrane of WT parasites and the parasitophorous vacuole (PV) membrane, with several points of close contact between the two membranes (Fig 9A). In contrast, macrophages infected for 72 hours with the two amastin knockdown clones contain amastigotes showing not only morphological alterations such as larger vesicles and partial disorganization of the sub-pellicular microtubule, but also striking variations in their contact with the macrophage PV membrane (Fig 9A and S5 Fig). Amastigote membranes from both RNAi knockdown clones present fewer regions of contact with the PV membrane than amastigote membranes from WT parasites. This difference observed in the interaction between the PV membrane and amastigotes of WT and RNAi knockdown parasites was quantified by measuring the total area of the PV and the area occupied by the parasite (Fig 9B). It should be noted that, in contrast to the PV membranes from macrophages infected with WT parasites, PV nembranes from macrophages infected with both δ-amastin knockdown parasites present regions where the lipid bilayer appears to be disrupted. Finally, it is also noteworthy that in a few images, including images of macrophage infected with RNAi knockdown clones, we observed two amastigotes in one PV, indicating that these parasites are able to divide within the PV (S5 Fig).

## Discussion

Expansion of families encoding surface proteins is one of the main characteristics revealed with the complete genome sequences of different trypanosomatid parasites. Several of these proteins appear to be involved with host parasite interactions and are likely to play a role in the parasite evasion of host immune responses. In the case of *T*. *cruzi* and *Leishmania*, gene families encoding surface proteins may be directly responsible for the ability of these parasites to invade and multiply within mammalian host cells. However, because they are encoded by multiple genes that are usually dispersed in the genome, knockout experiments to determine their functions may not be applied and, in the case of *T*. *cruzi* and most *Leishmania* species, the strategy of gene silencing has also been hampered by the absence of functional RNAi pathways in these parasites.

Amastins constitute a group of glycoproteins encoded by a multigene family present in the genome of several trypanosomatids. In spite of being thoroughly investigated in *T*. *cruzi* [7, 18], *L*. *major*, *L*. *infantum* [9] and *L*. *amazonensis* [10], a clear role for amastin genes has not been yet defined. In agreement with their proposed role related to parasite intracellular survival, comparative genomic studies have shown an increased amastin gene repertoire in most *Leishmania* species [12]. We showed here that, similar to other *Leishmania*, *L*. *braziliensis* has 52 amastin genes, with a vast majority (42 genes) belonging to the δ-amastin subfamily. It is noteworthy that *L*. *tarentolae*, a lizard parasite non-pathogenic to humans, which does not multiply intracellularly, has only 11 amastin genes and from those only 2 genes belong to the δ-amastin subfamily [30]. Since the increased amastin gene repertoire observed in *Leishmania* is due to the expansion of the δ-amastin subfamily, it is proposed that δ-amastins evolved novel functions related to intracellular survival of *Leishmania* spp within mammalian host macrophages [12].

Based on northern blot and RNA-seq data, most likely all δ-amastins are upregulated in *L*. *braziliensis* amastigotes. We also showed evidence indicating that all amastins are localized to the parasite surface. In an attempt to elucidate the role of δ-amastins, we took advantage of the fact that, different from *L*. *infantum* and other Old World *Leishmania* species, *L*. *braziliensis* has a functional RNAi pathway. Using a construct directing the expression of dsRNA containing sequences from one δ-amastin gene, we were able to evaluate the consequences on the intracellular survival of *L*. *braziliensis* amastigotes of knocking down expression of several genes of this amastin subfamily. The substantial decrease in intracellular parasite numbers after *in vitro* infection of mouse macrophages and the complete attenuated phenotype observed during *in vivo* infection with RNAi knockdown parasites revealed, as previously anticipated, an essential role of δ-amastins in a long-term infection of *Leishmania*.

Recent studies with different *T*. *cruzi* strains have also suggested a correlation between expression of δ-amastins and parasite virulence [31]. Different from most *T*. *cruzi* strains, the G strain exhibit very low infectivity both *in vitro* and *in vivo* [18]. Such lower infection capacity was associated with low transcript levels of δ-amastins, which are highly expressed in amastigotes in all *T*. *cruzi* strains analyzed, except in the G strain [18]. After transfecting the G strain with a vector that directs the constitutive expression of δ-amastins, an accelerated differentiation of amastigote into trypomastigotes was observed during late stages of the *in vitro* infection of HeLa cells. Moreover, in contrast to the infection of susceptible mouse strains with wild type G strain parasites, where amastigotes nests were observed only after day 5, amastigotes of G strain overexpressing δ-amastin were observed in mouse tissues at the third day after inoculation [31].

In contrast to *T*. *brucei*, in which RNAi has been used as a powerful tool for functional genomic studies, especially after the development of a system for tetracycline-regulated expression of dsRNAs [21] neither *T*. *cruzi* [20] nor Old World *L*. *major* and *L*. *donovani* [22] has functional RNAi machinery. The characterization of the complete RNAi pathway in *L*. *braziliensis*, including the identification of endogenous small interfering RNAs, or siRNAs and the proteins required for this pathway [4, 32] has allowed studies involving gene knockdown in this parasite and, most importantly, knocking down the expression of multigene families. RNA-seq analysis has shown that, besides reducing transcript levels of the target amastin gene, expression of dsRNA containing δ-amastin sequences affects other δ-amastin genes, while expression of amastin genes belonging to other subfamilies was not affected. This observation is highly relevant, since it allowed us to infer that the phenotypic differences observed in the knockdown parasites indeed resulted from specific reduced expression of δ-amastins. This prediction was confirmed by experiments testing the phenotype of parasites in which δ-amastin expression was knocked-down by RNAi and subsequently restored by transfecting a clone expressing amastin dsRNA with an RNAi-resistant amastin gene. The fact that we exclusively knocked-down δ-amastins also means that we need to test the function of members of other amastin subfamilies, which is currently underway.

The reduced numbers of intracellular amastigotes observed 48 and 72 hours after *in vitro* infection of mouse macrophages with parasites where δ-amastin expression has been knocked-down compared to WT parasites provided the first direct evidence of a role of δ-amastins related to parasite proliferation inside the macrophage PV. The fact that no significant differences were observed 24 hours post-infection also suggested that amastins do not play a significant role in the initial steps of parasite internalization, which occurs through receptor-mediated phagocytosis by the macrophages [33]. This observation also suggests that δ-amastins could be important for protection against the content of the PV and hence for survival but not directly for proliferation. Because, after *in vivo* infection of BALB/c mice, no living parasites were found in the footpads of these animals, it can be also speculated that, besides their role related to intracellular amastigote proliferation or survival in the PV, δ-amastins may also participate in mechanisms developed by the parasite to maintain the infection, such as evasion of host immune response. It should be noted that the role played by δ-amastin in *L*. *braziliensis* may be different from its role in *T*. *cruzi*, where overexpression of this gene did not affect intracellular multiplication of the G strain, but affects parasite differentiation from intracellular amastigote to infective trypomastigotes [31]. It is also noteworthy that, different from *Leishmania* spp, *T*. *cruzi* amastigotes multiply in the cytoplasm of a variety of mammalian cell types. Hence, since *T*. *cruzi* and *L*.*braziliensis* δ-amastins share 35% identity at the amino acid level, these proteins may have developed functions that are specific for the distinct intracellular niches these parasites occupy within their host cells [14].

Together with the phenotypic changes observed in the interaction of *L*. *braziliensis* with host macrophages, the similarity found between the 3D structures of amastins and mammalian claudins represents a beam of light shedded on the long lasting question regarding amastin gene function. In striking similarity to amastins, claudin proteins have four transmembrane domains, two extracellular hydrophilic domains with two highly conserved cysteine residues in their first hydrophilic domain. Amastins also present structural similarities with the adenylate cyclase (AC) family of proteins described in African trypanosomes [34], but in contrast to amastins, all members of the AC family localize to the flagellum of the parasite. As components of the apical intercellular seal in polarized epithelial cells formed by specific complexes named "tight junctions" [35], claudins form structures that not only serve as barriers, but also function to control paracellular channels, which are selectively permeable to ions and small charged molecules [27]. The close interaction between the *L*. *braziliensis* membrane and the PV membrane, also observed in macrophages from infected mouse ear tissues described by Zauli and colleagues [29] suggests a role of amastins in mediating this interaction. Images of macrophages infected with parasites with reduced δ-amastin expression showed not only that this interaction was drastically affected, but also that the structure of PV membrane bilayer became disorganized in the regions where there is no contact with the parasite membrane. Whether these changes in the interaction between the *Leishmania* and the PV membrane is the cause of the drastic reduction of parasite load observed in macrophages infected with amastin knockdown cell lines remains to be investigated. Although we have no direct evidence for the existence of an interaction between amastins and claudins, or with any other protein present in the PV membrane, it is reasonable to speculate that amastins may participate in the formation of a complex with proteins present in the macrophage PV membrane and that the formation of this complex may be essential for the survival of *Leishmania* inside the PV.

## Materials and Methods

### Ethics statement

All animal work was conducted according to national and international guidelines. Non infected and infected animals were kept with appropriate conditions of technical management in cages that were properly identified and sealed, preventing any contact with healthy animals. All animals were euthanized by cervical dislocation without sedation/anesthesia, before removal of footpad tissues.

### *In silico* analysis

Sequence analyses were performed using the *L*. *braziliensis MHOM/BR/75/M2904* genome database (ww.tritrypdb.orgw) to identify all amastin genes. Amastin sequences from *T*. *cruzi* [18] and *L*. *infantum* [9] retrieved from tritrypdb (www.tritrypdb.org) were used as queries in Blastp analyses (www.ncbi.nlm.nih.gov/blast/Blast.cgi). Multiple alignment of *L*. *braziliensis* amastin polypeptide sequences was done using the MUSCLE software (http://www.drive5.com/muscle/). Phylogenetic trees were constructed using a neighbor-joining algorithm with 1000 bootstrappings by MEGA 6 software [36].

PHYRE database was used to generate a predicted structural model. The protein sequence of amastins were obtained from the and submitted to Protein Homology/analogy Recognition Engine (PHYRE version 2) [24]. Based on homology sequence in PHYRE server, the three-dimensional structure of amastin was predicted. Homology modeling was performed using UCFS Chimera [37].

### Parasites cultures

Promastigote cultures of *Leishmania braziliensis* M2904 strain (MHOM/BR/75/M2904), kindly provided by Prof. Maria Norma Melo from the Parasitology Department at UFMG, were maintained by weekly passages in freshly prepared Schneider’s Insect Medium (Sigma-Aldrich Cat. No.S9895) supplemented with 10% heat-inactivated fetal bovine serum. To obtain axenic amastigotes, promastigotes cultures were incubated at 34°C in 100% FBS, 5%CO_2_ for 72 hours, as previously described [38].

### Plasmid constructs and parasite transfections

Plasmid constructs used to exogenously express in *L*. *braziliensis* short-hairpin RNAs targeting amastin mRNAs were derived from pIR1PHLEO plasmid [4], which was kindly provided by S. Beverley (Washington University). The vector pIR1PHLEO-Ama1060 was generated after inserted PCR products containing the coding region of amastin LbrM.20.1060 in the “antisense” followed by the “sense” orientation, with 200 pb fragment corresponding to the 5´UTR region between the coding sequences, to produce a stem-loop, into the XbaI site. After verifying the construct by restriction mapping and sequencing, 100 μg of the pIR1PHLEO-Ama1060 plasmid were digested with SwaI and the linear SSU-targeting fragment purified for transfection.

Stable transfections of *L*. *braziliensis* promastigotes were performed using a BioRad gene pulser. Parasites grown to mid-log phase were pelleted at 3000xg, washed once with cytomix electroporation buffer (120 mM KCl, 0.15 mM CaCl_2_, 10mM K2HPO_4_, 25 mM HEPES pH7.6, 2 mM EDTA and 5 mM MgCl_2_) and resuspended in cytomix buffer at a final concentration of 2x10^8^ cells/mL. Five hundred µL of cell suspension was mixed with 100μg of DNA in a 0.4 cm gap cuvette and submitted to two pulses of 1400 V (3.75 kV cm^-1^), 25 mF with a 10 s interval between pulses. Following electroporation promastigotes were grown in drug-free media overnight, and then plated on semisolid media [22] containing 2 mg/mL phleomycin (Sigma) and incubated at 34°C to generate clonal cell lines expressing the PHLEO marker. After colonies emerged (approximately, 2 weeks) they were recovered and grown to stationary phase in 1 mL Schneider’s Insect Medium and passaged thereafter in media containing 0.1 mg/mL phleomycin.

For cellular localization of GFP fusion proteins, sequences corresponding to the entire coding regions of three distinct amastin genes (LbrM.20.1060, LbrM.24.1600 and LbrM.30.0980) were PCR amplified from *L*. *braziliensis* genomic DNA using forward and reverse primers carrying XbaI restriction sites (see S1 Table). After digesting the amplicons with XbaI, they were inserted into the XbaI site of pSP72RαneoαGFP vector [28] in frame with the GFP coding region. A total of 100 μg of each plasmid construction was used to transfect *L*. *braziliensis* promastigotes as described above. Twenty four hours post-transfection, parasites were fixed with 2% paraformaldehyde for 30 min at 4°C and washed twice in phosphate-buffered saline (PBS 1X) at pH 7.4. After DNA staining with 1 μg/mL of 4’,6-diamidino-2-phenylindole (DAPI, Life Technologies), coverslips were mounted with ProLong Gold antifade reagent (Life Technologies). Images were acquired with a 100 x objective in the fluorescence microscope Nikon Eclipse Ti Tecnai G2-12 SpiritBiotwin FEI (120kV) at the Image Acquisition and Processing Center of ICB-UFMG.

Plasmids containing RNAi-resistant amastin sequences were constructed to rescue the RNAi phenotype, using DNA sequences generated synthetically by GenScript (http://www.genscript.com). Sequence encoding the amastin polypeptide LbrM.20.1060 was assembled with the third base modified in such a way that the amino acid was not altered as shown in the alignment of the WT LbrM.20.1060 amino acid sequence and the synthetic RNAi-resistant sequence (S2 Fig). In addition, an HA epitope tag was added to the position corresponding to the second hydrophilic region (amino acid position 131 and 149) and HindIII and BamHI restriction sites were added to the 5’ and 3’ ends, respectively to facilitate cloning into the pSP72RαneoαGFP plasmid. After replacing the GFP sequence by the synthetic gene into pSP72RαneoαGFP plasmid, transfection of promastigote cultures of WT parasites and from the RNAi1060-cl5 cell line with 100 μg of the circular plasmid was done by electroporation as described above. Following transfection, promastigotes were grown in drug-free medium over-night and plated on semi-solid media [21] containing 40 μg/mL of G418 (GIBCO) to select G418 resistant clones.

### Anti-amastin antibody preparation and western blot analyses

Total protein extracts were obtained by pelleting 10^6^ exponentially growing *L*. *braziliensis* promastigotes or *in vitro* derived amastigotes. Cell pellets were ressuspended in 100 μl of PBS and boiled before loading onto gel 12% SDS-PAGE. For western blotting, SDS-PAGE gels were transferred to membranes (Millipore) and the membranes were incubated with anti-HA antibodies (100 ng/mL, Sigma) or a polyclonal antibody raised against the recombinant amastin protein Ama::his1060 expressed in *E*. *coli* (immunized mouse sera diluted 1:100) for 1 hour at room temperature. The recombinant, his-tagged amastin was obtained by cloning in the pET-21(+) vector (Novagen) sequences corresponding to amino acids 30 to 78 obtained by PCR amplification of the amastin gene LbrM.20.1060. After primary antibody incubation, membranes were incubated with anti-mouse IgG (Sigma) peroxidase-conjugated secondary antibodies for 1 h at room temperature and revealed with the chemiluminescent substrate using the ECL kit (GE HealthCare).

### RNA preparation and northern blot analyses

Total RNA from *L*. *braziliensis* promastigotes and amastigotes were isolated using the Trizol reagent (Invitrogen). Northern blot analysis of total RNA separated on 1.2% agarose-formaldehyde gels was carried out as previously described [7]. All probes used in these studies were prepared by PCR amplification using specific primers for each amastin gene. To quantify the steady-state levels of the different *L*. *braziliensis* amastin transcripts in promastigotes and axenic amastigotes, we normalized the signals for each probe by hybridizing the same blots with a fragment corresponding to the 5S rRNA. Enrichment of small (< 200 nt) RNA fraction was carried out using the mirVanaTM miRNA Isolation Kit (Ambion) according the manufacturer’s procedure and resolved on a 15% Urea-polyacrylamide gel as previously described [17]. DNA oligonucleotide probes (corresponding to the forward strand of each element) were end-labelled with T4 polynucleotide kinase (NEB) and [γ-^32^P]-ATP and purified on a P6 column (Bio-Rad). Hybridizations were carried out in ExpressHyb solution (Clontech) overnight at 30°C. The membranes were exposed to X-ray films (Kodak) or revealed using the STORM840 PhosphoImager (GE HealthCare). To quantify the steady-state levels of the different *L*. *braziliensis* small RNAs present in promastigotes and axenic amastigotes, we normalized the signals after hybridizing the same blots with a probe corresponding to the glutamate-tRNA sequence.

### RNA-seq analyses

Total RNA was extracted from amastigotes obtained from WT cultures and cultures derived from the dsRNA expressing cell line RNAi1060-cl1. Two independent cDNA preparations for each RNA sample were carried out using with TruSeq RNA Library Prep Kit v2. Illumina next-generation sequencing was done using HiSeq (at BGI Hong Kong Tech Solutions) and MiSeq (at UFMG) following published methods. The quality of all RNA-Seq data was assessed using FastQC (http://www.bioinformatics.babraham.ac.uk/projects/fastqc/). Illumina adaptors were trimmed using Trimmomatic software (www.usadellab.org/cms/?page=trimmomatic). No additional processing of the primary data was required. After quality checking, paired end sequences were mapped to the *L*. *braziliensis* MHOM/BR/75/M290 genome V7.0 (www.tritryp.org) using TopHat version 2.0.11 [39], allowing up to 2 mismatches, 3 nucleotides of gap length and only unique hit per read. Results were piped through Samtools version 0.1.19 to sort by gene name, and stored in indexed BAM format. HTseq-count version 0.6.1 was used to count mapped read numbers for each gene. Statistical and differential expression analyses of the sample groups were undertaken by DESeq2 package [40] in the R environment, using linear models and empirical Bayes methods. Before quantile normalization and group-wise comparisons, the data were filtered to remove loci whose mean values were below the 10% quantile for all samples. Read depths were calculated by the mean of normalized counts of mapped reads. Sequence data was deposited at Sequence Read Archive (SRA) of the NCBI under SRA accession number SRP065143.

### *In vitro* infection

Primary BALB/c peritoneal macrophages (5×10^5^ adherent cells/well) were cultured in RPMI 1640 medium supplemented with 20% FBS, 2 mM L-glutamine, 200 U/mL penicillin G, and 100 μg/mL streptomycin sulfate, at pH 7.4, in 24-well culture plates with round glass coverslips within. To each well were added 5 x 10^6^ promastigotes in the stationary phase (five days) of *L*. *braziliensis* WT and mutant cells at a ratio of 10 parasites per macrophage in RPMI with 10% FBS. After 24 hours of infection, cells were washed with RPMI to remove parasites that were not internalized and maintained at different periods at 37°C. The slides were stained with 1 mg/mL of 49,6-diamidino-2-phenylindole (DAPI, Molecular Probes/Life Technologies) for 5 minutes and evaluated the degree of infection under a fluorescence microscope by counting the parasites in 100 infected cells. The experiments were performed in triplicate and the results were analyzed for significant differences using One Way ANOVA and Bonferroni’s Multiple Comparison.

### *In vivo* infection

Stationary phase promastigotes harvested on day 5 of culture were resuspended in PBS and injected subcutaneously in the hind footpad of 7-week old female BALB/c mice (4 mice per group) at 1 x10^7^ cells/mouse. One and 2 weeks post-infection, animals were sacrificed and their infected footpads were harvested for parasite quantification using the limiting-dilution assay. Cultures were examined under light microscopy for the presence of promastigotes 15 days after incubation at 25°C. Results were expressed as the negative logarithm titer of parasites corresponding to the last dilution where parasites were observed [41]. The statistical analysis of the *in vitro* and *in vivo* infectivity experiments was performed using the GraphPad Prism software (version 5.0 for Windows). Both in vitro and in vivo infection studies were carried out in strict accordance with the Brazilian laws regarding animal use (LEI N^°^11.794, DE 8 DE OUTUBRO DE 2008), all protocols being approved by the Committee on the Ethics of Animal Experiments of UFMG. Statistical analyzes were performed as indicated in the previous session.

### Electron microscopy analyses of macrophages infected by *L*. *braziliensis*

Electron microscopy analyses of macrophages infected by *L*. *braziliensis* were fixed in 5% glutaraldehyde in 0.1 M cacodylate buffer pH 7.2 and processed following standard protocols, including post-fixation in osmium tetroxide followed by block counterstaining with uranyl acetate and embedding in Epon resin [42]. Ultrathin sections were counterstaining with lead citrate and analyzed in the Transmission Electron Microscope Tecnai G2-12—SpiritBiotwin FEI—120 kV located at the Center of Microscopy at the Universidade Federal de Minas Gerais, Belo Horizonte, Brazil. The ratios between the areas of macrophage vacuoles and the areas of the amastigotes inside the vacuole were determined by measuring the areas of twenty vacuoles and their respective parasites from each experimental group using the Image J software. Kruskal-Wallis test followed by Dunn’s Multiple Comparison was performed to compare the three groups.

## Supporting Information

S1 Fig Multiple amino acid sequence alignment of *L*. *braziliensi (Lbr)*, *L*. *major* Friedlin (Lmjf), *L*. *infantum* (LinJ) amastin gene homologs. Alignments were performed using the ClustalW2 application. Identical amino acids are depicted with asterisks. Transmembrane regions are black highlighted (TM1-4) and the region from amino acids 52–62 corresponding to the amastin signature (C-[IVLYF]-[TS]-[LF]-[WF]-G-X-[KRQ]-X-[DENT]-C), conserved amongst all homologs amastin from *Leishmania* and *Trypanosoma* species, are highlighted in red.
(TIF)

S2 Fig Differential gene expression in WT parasites and parasites expressing δ-amastin dsRNA. Expression values are shown as log2 fold change.
(TIF)

S3 Fig Sequence comparison between *L*. *braziliensis* LbrM.20.1060 δ-amastin and the synthetic RNAi-resistant δ-amastin gene. Alignments of the two sequences show identical nucleotides depicted with asterisks and the region encoding the HA tag inserted in the second hydrophilic extracellular domain inside a red box.
(TIF)

S4 Fig PCR Detection of pIR1PHLEO-Ama1060 plasmid in transfected parasites. (A) The diagram shows the strategy used to knockdown δ-amastin, which resulted in the integration the pIR1PHLEO-Ama1060 plasmid into the SSU locus of the *L*. *braziliensis* genome. The linearized form of pIR1PHLEO-Ama1060, which drives the expression of amastin dsRNA, is shown below a schematic representation of the SSU locus. (B) The diagram shows the expected configuration of the SSU locus after homologous recombination of pIR1PHLEO-Ama1060 and the annealing positions of the primers used for PCR amplifications. (C) Agarose gel electrophoresis of PCR products that show integration of the pIR1PHLEO-Ama1060 plasmid in the *L*. *braziliensis* genome. Pairs of primers used in each PCR are indicated on the top of each gel and in the diagram shown in (B). The expected sizes (in base pairs, bp) of the amplicons are also indicated. PCR amplifications were done with DNA purified from promastigotes of (1) wild type *L*. *braziliensis* (WT), two cloned cell lines expressing amastin dsRNA ((2) RNAi-1060 cl1 and (3) RNAi-1060 cl5) and two cloned cell lines derived from RNAi-1060 cl5 that express the RNAi-resistant amastin sequence ((4) RNAi-R1 and (5) RNAi-R2). As a positive control for the amplification with the Phleo F and Phleo R primers and as a negative control for PCR with Phleo F and SSU R primers, we used pIR1PHLEO-Ama1060 plasmid DNA (6) as well as no DNA (7) in the reactions.
(TIF)

S5 Fig δ-amastin RNAi knocking down affects the contact between the amastigotes and the parasitophorous vacuole membranes. Transmission electron microscopy of macrophages infected with WT *L*. *braziliensis* (WT) and two cloned cell lines expressing amastin siRNA, RNAi-1060 cl1 and RNAi-1060 cl5. Note the tight contact between the amastigotes and PV membranes in WT parasites and a greater distance between the parasite and the PV membrane in macrophages infected with RNAi knockdown parasites.
(TIF)

S1 Table Differentially expressed amastin genes between WT amastigotes and RNAi knockdown amastigotes. (DOCX)
