# Supplementary figures and images for: Amastin Knockdown in Leishmania braziliensis Affects Parasite-Macrophage Interaction and Results in Impaired Viability of Intracellular Amastigotes
Source: PLoS Pathog. 2015 Dec 7;11(12):e1005296. doi: 10.1371/journal.ppat.1005296 (PMC4671664; doi:10.1371/journal.ppat.1005296)

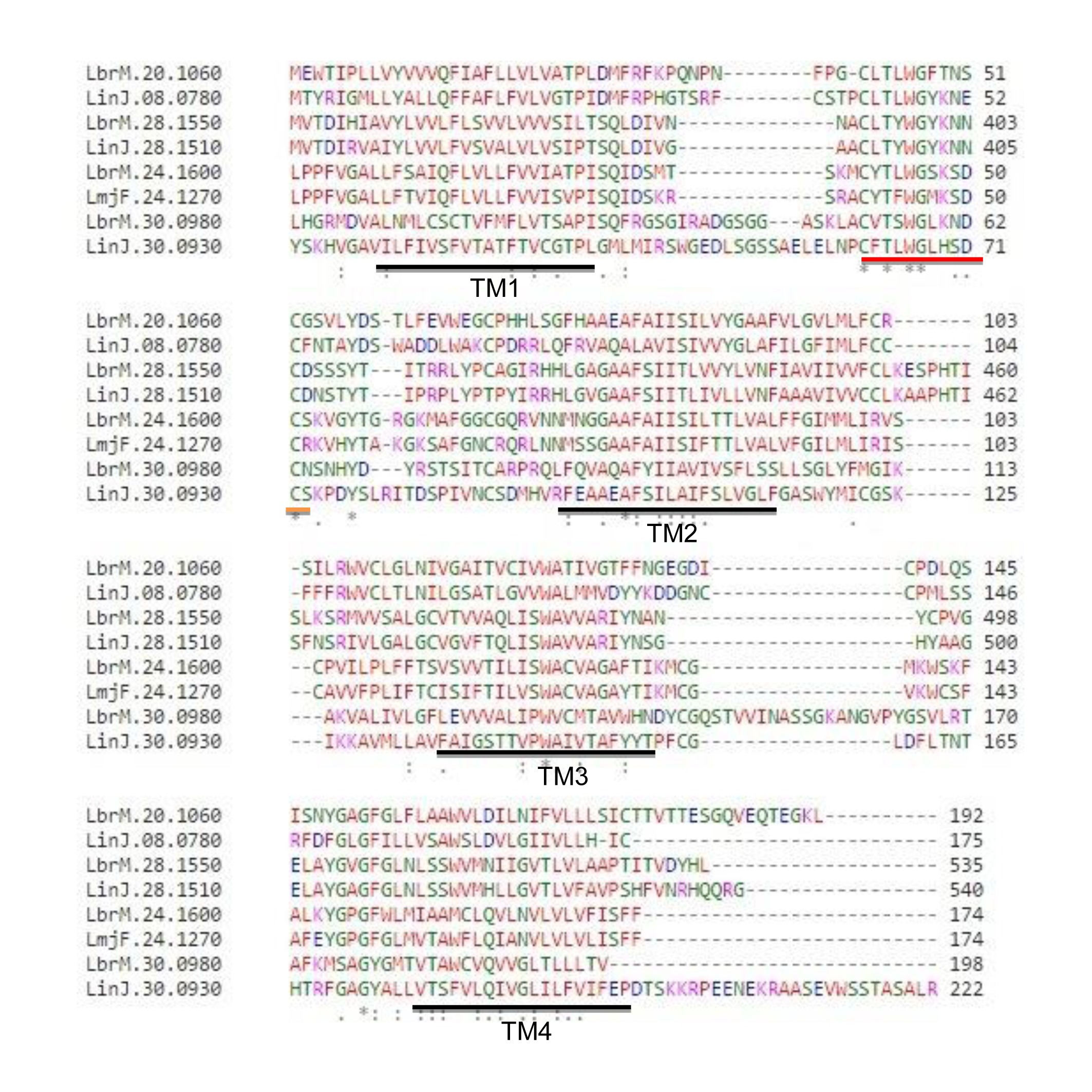

Supplement: S1 Fig — Alignments were performed using the ClustalW2 application. Identical amino acids are depicted with asterisks. Transmembrane regions are black highlighted (TM1-4) and the region from amino acids 52–62 corresponding to the amastin signature (C-[IVLYF]-[TS]-[LF]-[WF]-G-X-[KRQ]-X-[DENT]-C), conserved amongst all homologs amastin from Leishmania and Trypanosoma species, are highlighted in red. (TIF) [file ppat.1005296.s001.tif]

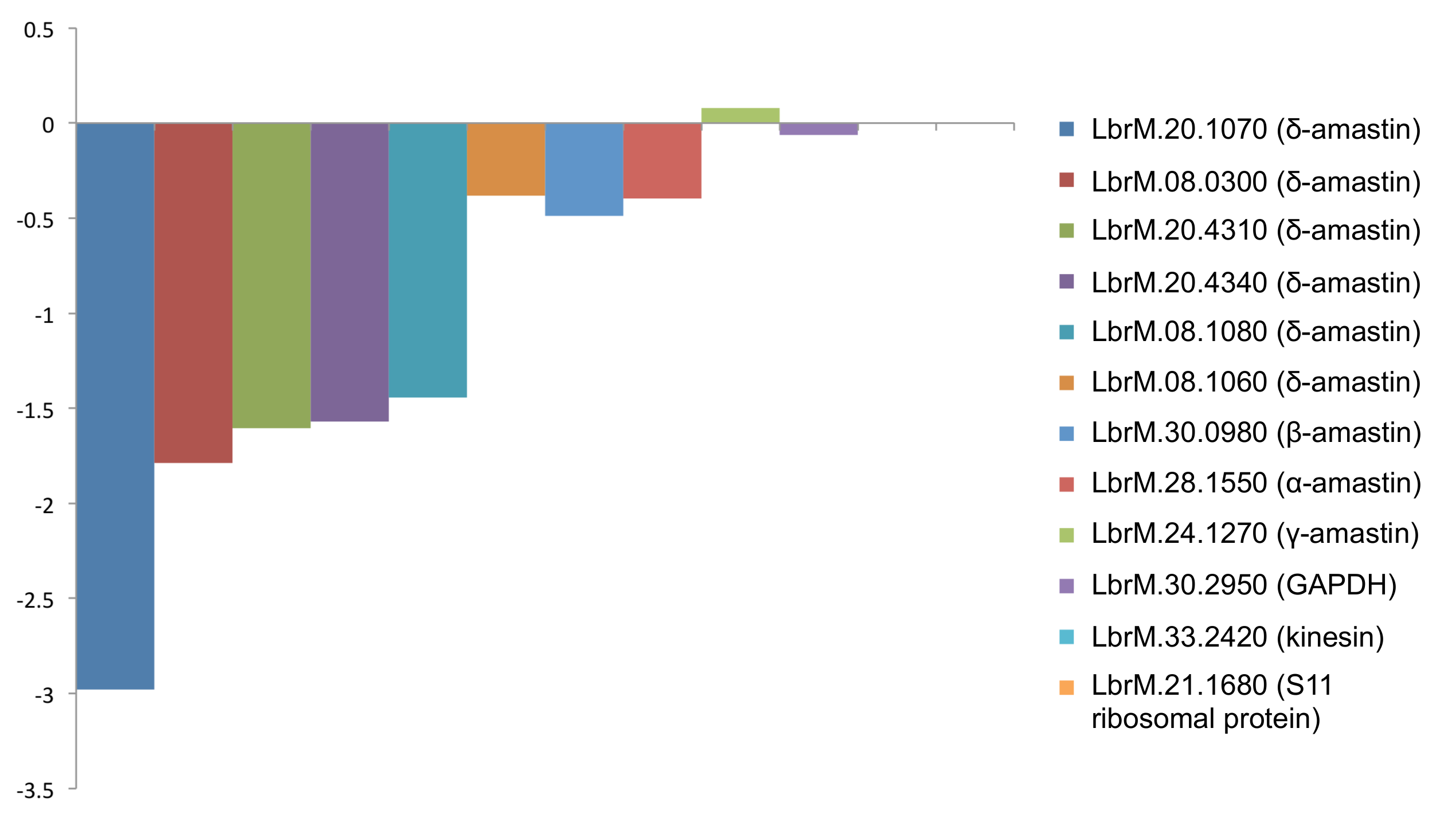

Supplement: S2 Fig — Expression values are shown as log2 fold change. (TIF) [file ppat.1005296.s002.tif]

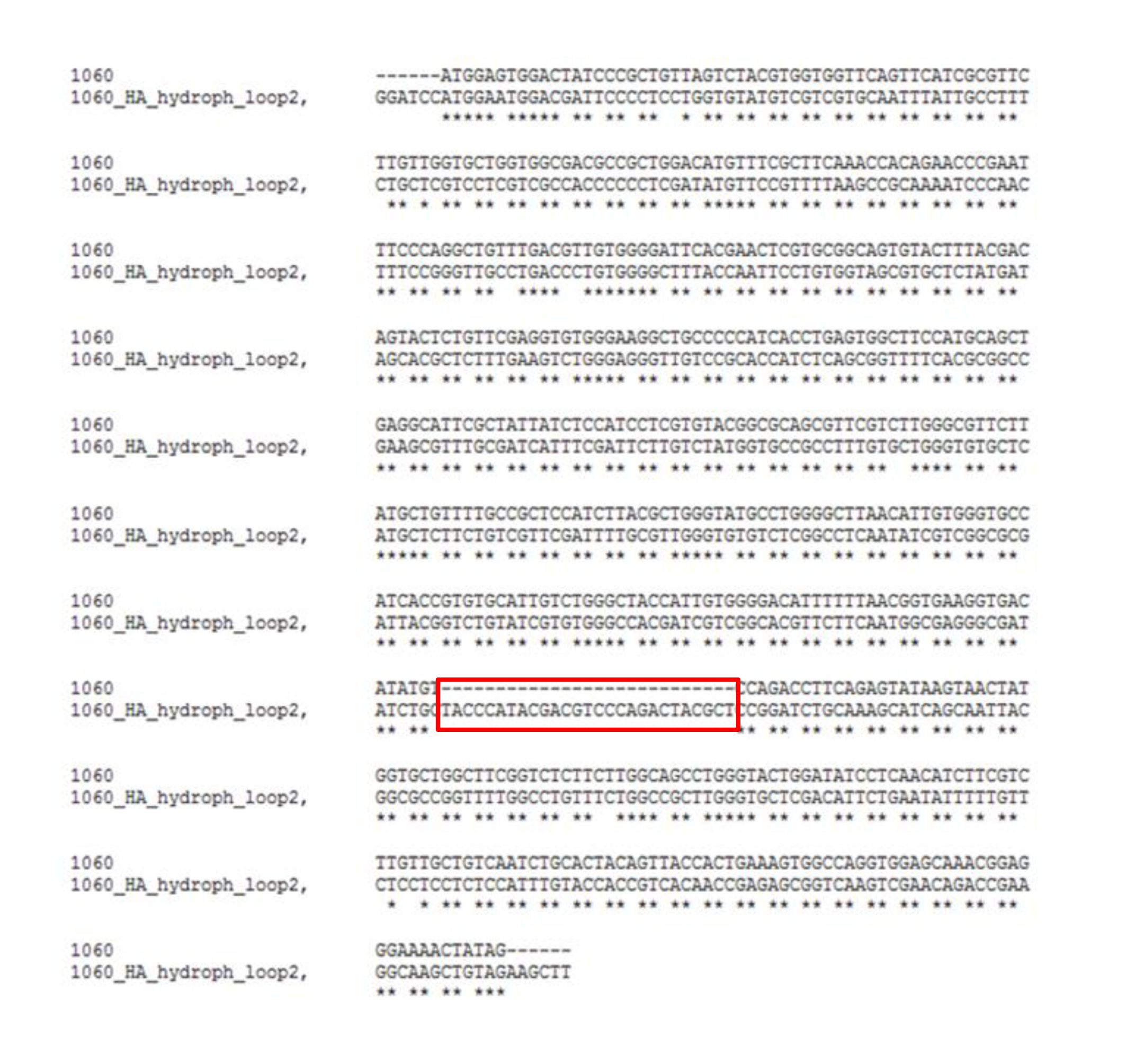

Supplement: S3 Fig — Alignments of the two sequences show identical nucleotides depicted with asterisks and the region encoding the HA tag inserted in the second hydrophilic extracellular domain inside a red box. (TIF) [file ppat.1005296.s003.tif]

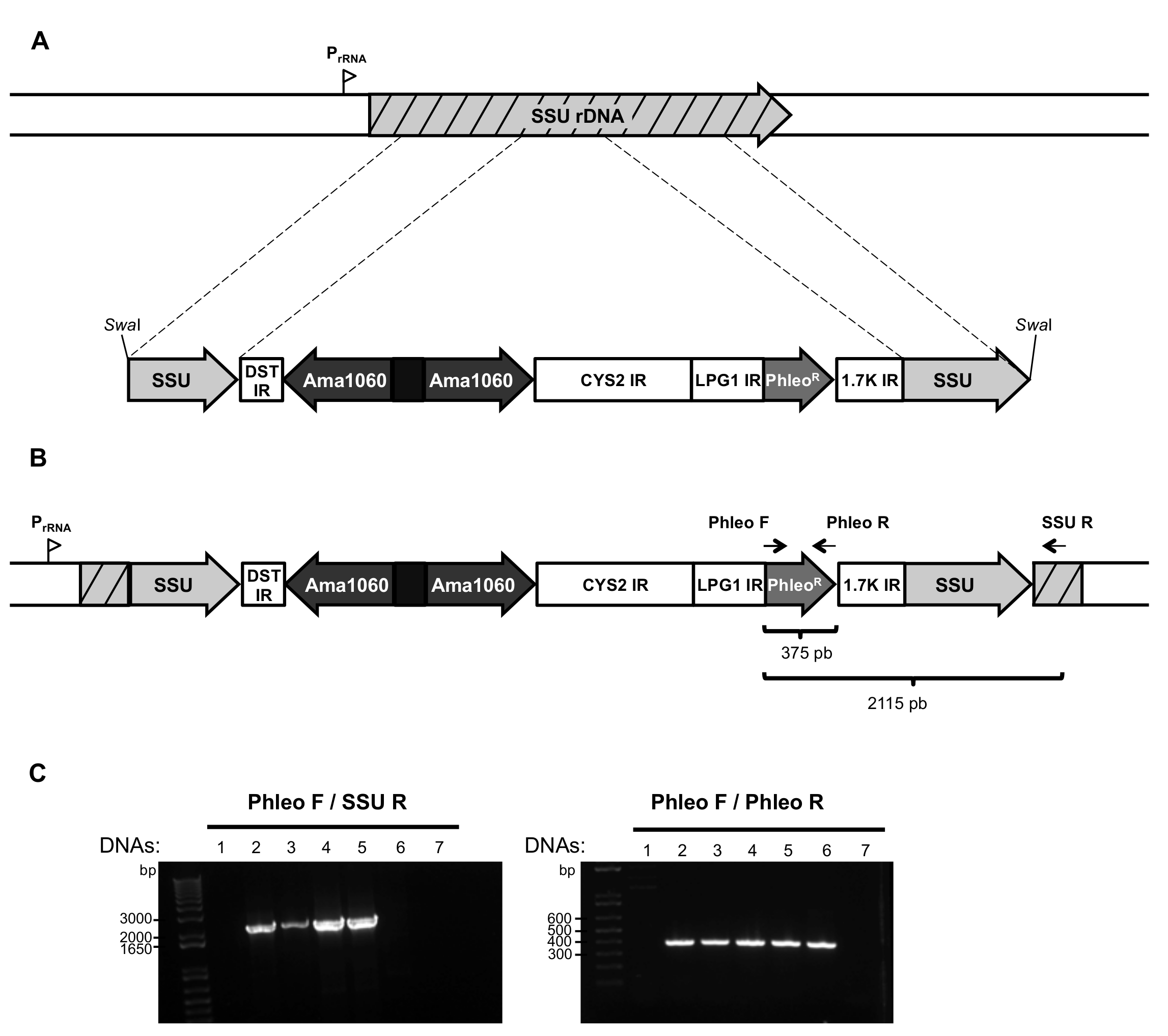

Supplement: S4 Fig — (A) The diagram shows the strategy used to knockdown δ-amastin, which resulted in the integration the pIR1PHLEO-Ama1060 plasmid into the SSU locus of the L. braziliensis genome. The linearized form of pIR1PHLEO-Ama1060, which drives the expression of amastin dsRNA, is shown below a schematic representation of the SSU locus. (B) The diagram shows the expected configuration of the SSU locus after homologous recombination of pIR1PHLEO-Ama1060 and the annealing positions of the primers used for PCR amplifications. (C) Agarose gel electrophoresis of PCR products that show integration of the pIR1PHLEO-Ama1060 plasmid in the L. braziliensis genome. Pairs of primers used in each PCR are indicated on the top of each gel and in the diagram shown in (B). The expected sizes (in base pairs, bp) of the amplicons are also indicated. PCR amplifications were done with DNA purified from promastigotes of (1) wild type L. braziliensis (WT), two cloned cell lines expressing amastin dsRNA ((2) RNAi-1060 cl1 and (3) RNAi-1060 cl5) and two cloned cell lines derived from RNAi-1060 cl5 that express the RNAi-resistant amastin sequence ((4) RNAi-R1 and (5) RNAi-R2). As a positive control for the amplification with the Phleo F and Phleo R primers and as a negative control for PCR with Phleo F and SSU R primers, we used pIR1PHLEO-Ama1060 plasmid DNA (6) as well as no DNA (7) in the reactions. (TIF) [file ppat.1005296.s004.tif]

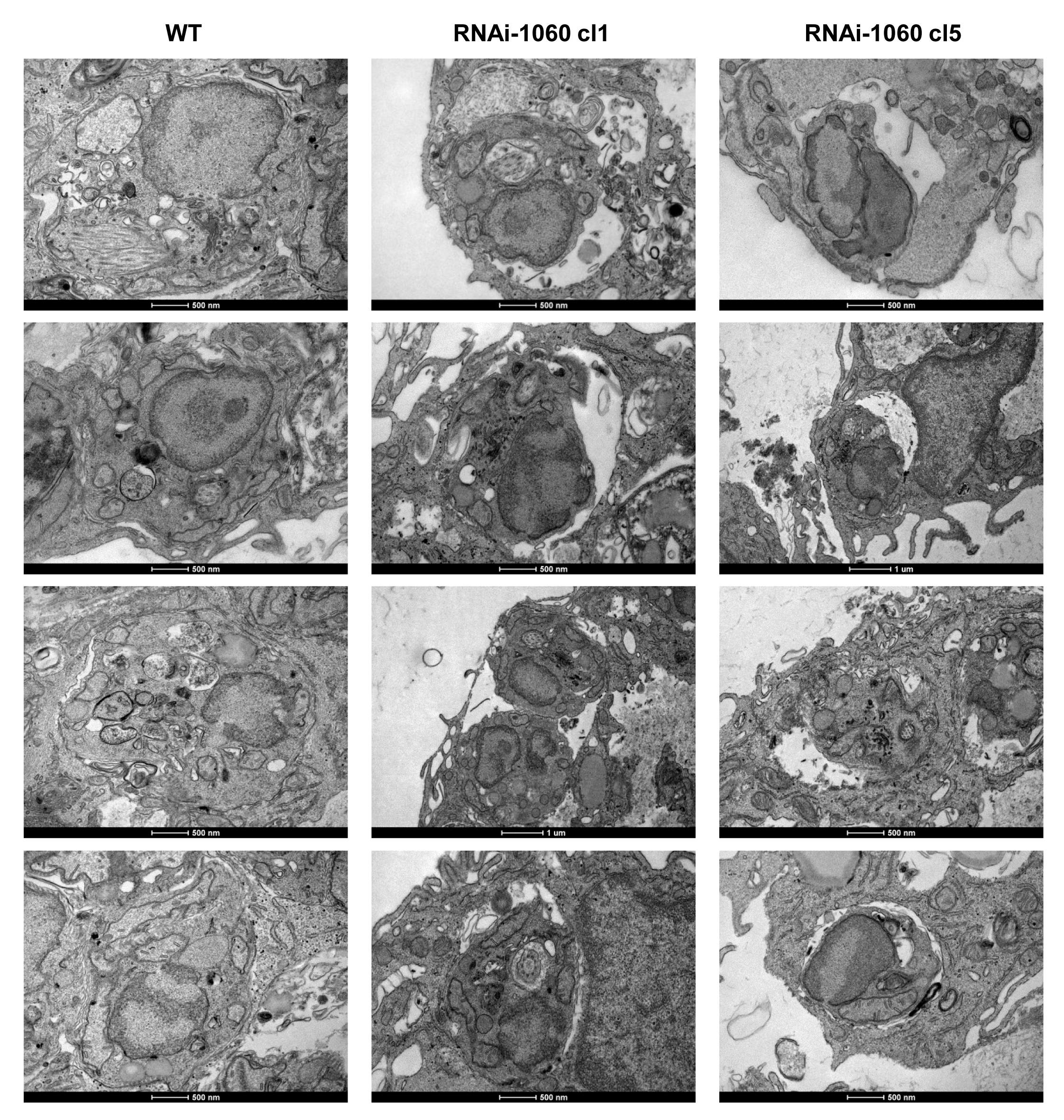

Supplement: S5 Fig — Transmission electron microscopy of macrophages infected with WT L. braziliensis (WT) and two cloned cell lines expressing amastin siRNA, RNAi-1060 cl1 and RNAi-1060 cl5. Note the tight contact between the amastigotes and PV membranes in WT parasites and a greater distance between the parasite and the PV membrane in macrophages infected with RNAi knockdown parasites. (TIF) [file ppat.1005296.s005.tif]
