# Supplementary material for: Amastin Knockdown in Leishmania braziliensis Affects Parasite-Macrophage Interaction and Results in Impaired Viability of Intracellular Amastigotes
Source: PLoS Pathog. 2015 Dec 7;11(12):e1005296. doi: 10.1371/journal.ppat.1005296 (PMC4671664; doi:10.1371/journal.ppat.1005296)
Supplement: S1 Table — (DOCX) [file ppat.1005296.s006.docx]

| Gene ID | Description | log2FoldChange | P value |
| --- | --- | --- | --- |
| LbrM.08.0300 | δ-amastin | -1.788 | 0.003 |
| LbrM.08.0290 | δ-amastin | -1.647 | 0.006 |
| LbrM.08.0310 | δ-amastin | -1.537 | 0.01 |
| LbrM.08.1050 | δ-amastin | 0.305 | 0.609 |
| LbrM.08.1060 | δ-amastin | -0.382 | 0.513 |
| LbrM.08.0670 | δ-amastin | -0.919 | 0.204 |
| LbrM.08.0680 | δ-amastin | -2.165 | 0.001 |
| LbrM.08.0990 | δ-amastin | -1.936 | 0.002 |
| LbrM.08.1030 | δ-amastin | -0.276 | 0.644 |
| LbrM.08.1040 | δ-amastin | -1.312 | 0.027 |
| LbrM.08.1080 | δ-amastin | -1.443 | 0.017 |
| LbrM.08.1090 | δ-amastin | -1.492 | 0.013 |
| LbrM.08.1100 | δ-amastin | -0.757 | 0.204 |
| LbrM.08.1110 | δ-amastin | -1.1 | 0.153 |
| LbrM.08.1120 | δ-amastin | -0.826 | 0.207 |
| LbrM.08.1130 | δ-amastin | -1.997 | 0.001 |
| LbrM.08.1140 | δ-amastin | -1.899 | 0.007 |
| LbrM.10.1520 | δ-amastin | -0.741 | 0.238 |
| LbrM.13.1330 | δ-amastin | -0.451 | 0.531 |
| LbrM.14.0500 | δ-amastin | 0.595 | 0.328 |
| LbrM.14.0490 | δ-amastin | -0.725 | 0.248 |
| LbrM.18.0460 | δ-amastin | -1.085 | 0.069 |
| LbrM.18.0470 | δ-amastin | -1.16 | 0.051 |
| LbrM.20.1060 | δ-amastin | -2.866 | 0 |
| LbrM.20.1070 | δ-amastin | -2.979 | 0 |
| LbrM.20.1080 | δ-amastin | -2.821 | 0 |
| LbrM.20.4340 | δ-amastin | -1.57 | 0.018 |
| LbrM.20.4310 | δ-amastin | -1.604 | 0.017 |
| LbrM.20.0780 | δ-amastin | -0.999 | 0.096 |
| LbrM.20.0800 | δ-amastin | -1.047 | 0.084 |
| LbrM.20.1090 | δ-amastin | -0.963 | 0.102 |
| LbrM.20.0950 | δ-amastin | -0.947 | 0.275 |
| LbrM.20.0790 | δ-amastin | -0.648 | 0.273 |
| LbrM.20.0960 | δ-amastin | 1.787 | 0.188 |
| LbrM.20.1480 | δ-amastin | -1.932 | 0.003 |
| LbrM.20.2370 | δ-amastin | -0.086 | 0.895 |
| LbrM.20.2410 | δ-amastin | -1.365 | 0.026 |
| LbrM.20.2870 | δ-amastin | 0.205 | 0.739 |
| LbrM.20.4290 | δ-amastin | -1.76 | 0.004 |
| LbrM.20.4300 | δ-amastin | -1.668 | 0.008 |
| LbrM.20.4320 | δ-amastin | -1.877 | 0.004 |
| LbrM.24.1270 | γ-amastin | 0.079 | 0.901 |
| LbrM.24.1280 | γ-amastin | 0.598 | 0.416 |
| LbrM.24.1600 | γ-amastin | 0.667 | 0.361 |
| LbrM.24.1610 | γ-amastin | 0.78 | 0.189 |
| LbrM.24.1590 | γ-amastin | 1.09 | 0.142 |
| LbrM.24.1290 | γ-amastin | 1.478 | 0.037 |
| LbrM.28.1550 | α-amastin | -0.396 | 0.552 |
| LbrM.28.1540 | α-amastin | -0.3 | 0.676 |
| LbrM.30.0980 | β-amastin | -0.488 | 0.409 |
| LbrM.30.1000 | β-amastin | 0.302 | 0.626 |
| LbrM.30.2950 | GAPDH | 0.958 | 0.919 |
| LbrM.33.2420 | Kinesin | 0 | 1 |
| LbrM.21.1680 | S11 ribosomal p | 0 | 1 |
